# Self-assembled [2 + 3] organic-imine cage as an artificial light harvester for the photocatalytic organic transformation in an aqueous medium

**DOI:** 10.1039/d5ra07378b

**Published:** 2025-11-07

**Authors:** Chanchala Kumari, Atul Kumar

**Affiliations:** a Department of Chemistry, Birla Institute of Technology Mesra Ranchi-835215 India atulkumar@bitmesra.ac.in

## Abstract

Designing an efficient visible light-mediated photocatalyst based on artificial light harvesting systems (LHSs) contemporary to photosynthetic complexes provides a powerful approach for energy capture and conversion. Herein, we report the synthesis of a supramolecular [2 + 3] organic-imine cage CA1*via* dynamic covalent amine condensation of a triazine-based tri-aldehyde and *cis*-(1*R*,2*S*)-cyclohexyl-diamine. The designed cage CA1 features an electron-deficient interior cavity that efficiently encapsulates the aggregation-induced emissive (AIE) tetraphenylethylene (TPE) fluorophore. Encapsulation of TPE in a cage complex suppresses non-radiative decay and yields intense blue emission. The host–guest system CA1⊃TPE exhibits a 10-fold emission enhancement due to the AIE effect in the aggregate state (90% water/THF solution) with a large stokes shift. The morphological characterization of the aggregate is well established through SEM and DLS analysis, revealing the formation of spherical nano-aggregate particles. Blue emissive nano-aggregate of CA1⊃TPE exhibits strong spectral overlap with Rhodamine B (RhB) and is utilised for the fabrication of artificial LHSs by Förster resonance energy transfer (FRET) between energy donor CA1⊃TPE and energy acceptor RhB in the 90% water/THF mixture. The FRET system CA1⊃TPE@RhB exhibits a maximum energy efficiency of 88.9% and an antenna effect of 11 at a 10 : 1 donor:acceptor ratio. The light-harvester complex CA1⊃TPE@RhB is further utilised as a visible light-driven photocatalyst for the condensation reaction involving substituted benzaldehyde and malononitrile in a green aqueous environment, demonstrating an improved yield compared to CA1⊃TPE and RhB alone.

## Abstract 2

A nano-aggregate of CA1⊃TPE forms a FRET pair with Rhodamine B to facilitate the photocatalysis of substituted benzaldehyde and malonitrile in water.

## Graphical abstract

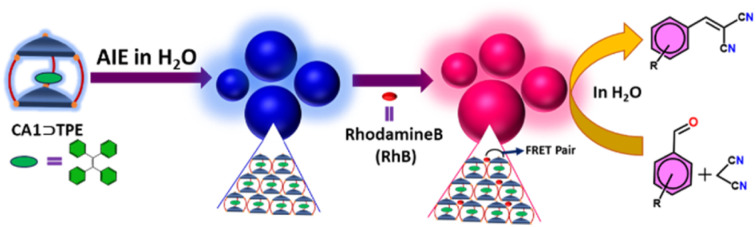

## Introduction

Natural photosynthesis is one of the most elegant and efficient solar energy conversion systems found in nature, in which excitation energy is captured and channelled by antenna protein complexes embedded in dense arrays of chlorophyll pigments and transferred *via* an energy transfer process to the carotenoid reaction centre, where it drives critical photochemical reactions.^1–7^ The development of artificial LHSs capable of efficiently mimicking the natural photosynthetic process has garnered considerable attention in recent years for the development of sustainable energy materials.^8–14^ Chemists have endeavoured to replicate natural photosynthetic processes to create artificial light-harvesting systems (LHSs), which serve as a sustainable clean energy source for various applications in the field of optoelectronic materials, optical sensor, bio-imaging, and photocatalysis.^15–20^ The development of photoactive supramolecular materials through self-assembly of functionalized molecular subunits offers an alternative approach to designing artificial LHSs using fluorescence resonance energy transfer (FRET).^21–28^ A fundamental requirement for the design of artificial light-harvesting systems (LHSs) through energy transfer from the donor to the acceptor *via* the FRET process includes the necessity for an appropriate Förster distance between the acceptor and donor, which mandates an overlap between the absorbance spectrum of the acceptor and the emission spectrum of the donor, and the construction of a collection of energy donor chromophores arranged in a densely packed configuration (spatial separation to fall within 1–10 nm) to capture light energy efficiently and function as an antenna.^29–32^ To replicate this function synthetically, researchers have designed a scaffold's structure that can precisely organise organic chromophores inside its cavity while preventing aggregation-caused quenching (ACQ), which is a common challenge in conventional systems.^33–36^ Inspired by the natural photosystem, synthetic chemists have explored diverse strategies for constructing artificial LHSs using conjugated polymers, dendrimers, porphyrin arrays, peptides, and other materials.^37–41^ Despite this, most artificial LHSs are made up of tedious multi-step covalent synthesis. In this regard, dynamic covalent chemistry (DCC) is a complementary approach to designing three-dimensional supramolecular organic covalent cages using an imine-condensation reaction in single-pot synthesis.^42–47^ The DCC approach enables reversible bond formation that allows error correction during self-assembly, resulting in the formation of the single most stable structurally defined thermodynamic product in high yield. The topologies of these structures can be adjusted through the meticulous choice of symmetry and connectivity of the building blocks, resulting in tetrahedral, cubic, and other intricate geometries that feature rigid, shape-persistent, well-defined, and customizable internal cavities. These cavities are capable of encapsulating guest molecules in contrast to extended porous networks, such as covalent organic frameworks (COFs) or metal–organic frameworks (MOFs).^48–54^ Owing to their inherent microporosity and adjustable functional groups, organic cages have been explored for applications in gas storage and separation, heterogeneous catalysis, molecular recognition, *etc.*^55–57^ Although organic cages constructed *via* DCC have shown success in host–guest chemistry, their applications in photophysical systems, especially in aqueous systems, remain limited due to difficulties in achieving both water solubility and strong photoluminescence. The self-assembled supramolecular organic cages function as molecular hosts, creating confined environments that allow donor–acceptor pairs to remain in close proximity, thereby enabling an efficient short-range through-space energy transfer process essential for the design of artificial LHSs.^58–64^ Furthermore, to mitigate the impact of the ACQ effect in the aggregate state, the incorporation of aggregation-induced emissive (AIE) fluorophores, such as tetraphenylethylene (TPE), as guest molecules within supramolecular systems has paved the way for the development of highly efficient light-harvesting systems (LHSs) that function effectively in the aggregate state.^65–69^ TPE is weakly emissive in dilute solutions due to free intramolecular rotations but exhibits strong fluorescence in the aggregate or confined state due to restricted intramolecular motions (RIM).^70–74^ This AIE behaviour complements the nature of supramolecular aggregation or encapsulation, allowing for enhanced emission and signal amplification. The incorporation of an AIE active molecule within the supramolecular framework provides a powerful approach toward the construction of highly emissive energy donor species that can transfer energy to suitable energy acceptors for generating artificial LHSs by the FRET process.^75–84^ Recent studies have successfully used these AIEgens into coordination architectures of metallacages and metallacycles, resulting in brightly emissive donor assemblies.^85–94^ Therefore, the development of artificial LHSs utilising covalent organic cages that maintain structural integrity, circumvent the ACQ effect, exhibit high energy efficiency and an antenna effect in green aqueous environments, and facilitate photocatalytic reactions through energy output under mild conditions remains a considerable challenge.

In this context, we report a triazine-based supramolecular organic-imine cage CA1 synthesized using a dynamic covalent approach, which possesses a well-defined electron-deficient cavity that encapsulates an AIE-active TPE molecule and forms a host–guest pair CA1⊃TPE. The CA1⊃TPE in the 90% H_2_O/THF mixture forms a spherical nano-aggregate and exhibits strong blue emission with a large Stokes shift due to the AIE effect. The host–guest complex CA1⊃TPE in the aggregate state shows considerable overlap of its emission with the absorbance of Rhodamine (RhB) and thus acts as an efficient platform for fabricating artificial LHSs by the FRET process, where CA1⊃TPE acts as an energy donor and Rhodamine (RhB) acts as an energy acceptor. Engineered artificial LHS CA1⊃TPE + RhB in the aggregate state is also used as a photocatalyst. Here, the output energy is utilized for a condensation reaction driven by visible light, involving substituted benzaldehyde and malononitrile in an aqueous environment. Overall, this study emphasises the supramolecular encapsulation of AIE fluorophores within a covalent organic-imine cage, which is aimed at developing artificial LHSs that facilitate organic transformations mediated by visible light under environmentally friendly aqueous conditions.

## Experimental section

### Materials and methods

The chemicals and solvents used in the present study were purchased from commercial sources and were used without further purification. NMR spectra were recorded in a 400 MHz NMR spectrophotometer (JEOL, Japan; JNM ECZ400S/LI), and the chemical shifts (*δ*) in the ^1^H NMR spectra are reported in ppm relative to tetramethylsilane (Me_4_Si) as an internal standard (0.0 ppm) or proton resonance resulting from the incomplete deuteration of the solvents CDCl_3_ (7.26 ppm). ^13^C NMR spectra were recorded at 100 MHz, and the chemical shifts (*δ*) were reported in ppm relative to external CDCl_3_ at 77.8–77.2 ppm. Electrospray ionization mass spectrometry (ESI-MS) experiments were carried out using an Agilent 6538 Ultra-High Definition (UHD) Accurate Mass Q-TOF spectrometer with standard spectroscopic-grade solvents. FTIR spectra were recorded using an FTIR spectrometer, the ALPHA II model of Bruker, Germany. An Analab μ-ThermoCal10 instrument was used for melting point determination. A UV-visible spectrophotometer (UV 3200) from LABINDIA Analytical was used to record the UV-visible spectra. The steady-state and time-resolved PL spectra were recorded using an FLS1000 PL spectrometer from Edinburgh Instruments Ltd, UK. The time-resolved PL decay profiles were measured after excitation with a 405-nm picosecond pulsed laser source. The integrated sphere setup was used to measure the absolute quantum yield of CA1⊃TPE@RhB and CA1⊃TPE. The 1931 Commission Internationale de l’Eclairage (CIE) chromaticity coordinates for the corresponding photoluminescence spectra were calculated using “GOCIE” software. A field emission scanning electron microscopy (FESEM) image was obtained using a ZEISS SIGMA-300 FESEM instrument by depositing the sample on a glass wafer. Dynamic light scattering (DLS) measurements were performed using a Zetasizer Nano Series Nano-ZS instrument, Malvern. THF/water mixtures with various water fractions were prepared by slowly adding ultrapure water into the THF solution of the samples. A Philips 50 W LED bulb (5000 Lm, 7500K) was used as a white light source for catalysis.

### Synthesis of ligand L

In a 250 mL round-bottom flask, 4-hydroxybenzaldehyde (3.97 g, 32.50 mmol) and sodium hydroxide (1.30 g, 32.5 mmol) were dissolved in 100 mL of water, and the reaction mixture was stirred at room temperature for 1 h. In a separate conical flask, cyanuric chloride (2 g, 10.84 mmol) was dissolved in 150 mL of acetone, and the solution was added dropwise to the reaction mixture at room temperature. The reaction mixture was refluxed for 24 h. The progress of the reaction was monitored using thin-layer chromatography. After the completion of the reaction, acetone was evaporated; subsequently, water was added and extracted with chloroform. The crude product was purified by column chromatography using silica gel (EtOH : hexane) as a white solid. Yield: 2.30 g (96%). ^1^H NMR (400 MHz; CDCl_3_) *δ* (ppm): 9.99 (s, 1H), 7.91 (d, 2H, *J* = 8 Hz), 7.31 (d, 2H, *J* = 8 Hz). ^13^C NMR (100 MHz, CDCl_3_) *δ* (ppm): 190.6, 173.6, 155.8, 134.8, 131.6, 122.3. FT-IR, *ν* (cm^−1^): 1699, 1565, 1365, 1209, 841. ESI MS (*m*/*z*): for [M + Na]^+^ is 464.39.

### Synthesis of cage CA1

In a 250 mL round-bottom flask, triazine-based *tri*-aldehyde ligand L (15 mg, 0.033 mmol) was dissolved in 60 mL CHCl_3_. In a separate conical flask, (1*R*,2*S*)-*cis*-cyclohexane-1,2-diamine (5.82 mg, 0.050 mmol) was dissolved in 30 mL CHCl_3,_ and this solution was added dropwise using a dropping funnel to the stirring solution of the ligand at room temperature. Then, the reaction mixture was stirred for an additional 48 h at room temperature. The progress of the reaction (the consumption of the ligand) was monitored using thin-layer chromatography. After completion of the reaction, the solvent was removed, and the crude solid was washed several times with methanol, which gave the product as a white solid. Yield: 7 mg (24%). ^1^H NMR (400 MHz, CDCl_3_) *δ* (ppm): 8.64 (12H, s), 7.52 (d, 12H, *J* = 16 Hz), 5.67(6H, s), 3.09(12H), 2.74(12H). FTIR, *ν* (cm^−1^): 3642, 2957, 1436, 1229, 1156. ^1^H DOSY NMR (400 MHz, CDCl_3_): log *D* = −9.38. ESI MS (*m*/*z*): For [M + 2H]^2+^ 559.14, and 1118.28 for [M + H]^+^.

### Encapsulation of TPE inside cage CA1

The encapsulation of TPE inside cage CA1 was achieved *via* a host–guest complexation process. In the first step, both cage and TPE were dissolved separately in CDCl_3_. Then, the cage solution was titrated with TPE by gradually adding the TPE solution and stirring for 5 min at room temperature. ^1^H NMR, DOSY NMR and ESI-MS confirmed the host–guest complex formation. ^1^H NMR (400 MHz, CDCl_3_) *δ* (ppm): 8.64 (12H, s), 7.52 (d, 12H, *J* = 16 Hz), 5.67(6H, s),3.09(12H, s), 2.74(12H, s). ^1^H DOSY NMR (400 MHz, CDCl_3_): log *D* = −9.74. ESI MS (*m*/*z*): For [M + 2H]^2+^ 725.86, and 1450.73 for [M + H]^+^.

### Fluorescence average lifetime (τ_av_) calculation

As obtained by bi-exponential fitting, *τ*_av_ is calculated using (eqn (1)):1
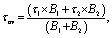
where *τ*_1_ and *τ*_2_ represent the lifetimes with amplitudes of *B*_1_ and *B*_2_, respectively.

### Energy transfer efficiency (*Φ*_ET_) calculation

Energy transfer efficiency is calculated using (eqn (2)):2
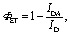
where *I*_DA_ = intensity of donor emission in the presence of acceptor, *I*_D_ = intensity of donor emission in the absence of acceptor and *Φ*_ET_ = fractional energy transfer efficiency.

### Antenna effect

The antenna effect calculated for a certain concentration of donor and acceptor is equal to the ratio of emission intensity at 577 nm (eqn (3)) upon excitation of the donor.3
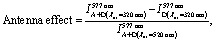
where 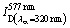
*=* emission intensity of donor (10^−5^M [CA1⊃TPE]) at 577 nm upon excitation at 320 nm; 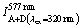 = emission intensity of (donor + acceptor) {10^−5^M [CA1⊃TPE] + 10^−6^ M[RhB]} at 577 nm upon excitation at 320 nm; and 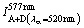 = emission intensity of (donor + acceptor) 10^−5^M [CA1⊃TPE] + 10^−6^ M[RhB]} at 577 nm upon excitation at 520 nm.

### General procedure for the synthesis of 7a–j

In a 25 mL round-bottom flask, the benzaldehyde derivative (0.97 mmol) and malononitrile (1.95 mmol) were suspended in 2 mL of water. To this solution, LHS-based photocatalyst CA1⊃TPE@RhB (CA1⊃TPE@ 7.5 mol% and RhB@1.5 mol% in 90% water/THF) was added to the reaction mixture. Then, the reaction was continuously stirred at 50 °C for 1 h in the presence of air under visible light. After completion, the final compound was extracted with ethyl acetate and water, which gave a white crystalline product.

7a: ^1^H NMR (400 MHz; CDCl_3_) *δ* (ppm): 7.91 (d, 2H, *J* = 8 Hz), 7.78 (s, 1H), 7.64 (m, 1H), 7.55 (m, 2H); 7b: ^1^H NMR (400 MHz; CDCl_3_) *δ* (ppm): 7.81 (d, 2H, *J* = 8 Hz), 7.72 (s, 1H), 7.34 (d, 2H, *J* = 8 Hz), 2.46 (s, 3H); 7c: ^1^H NMR (400 MHz; CDCl_3_) *δ* (ppm): 7.91 (d, 2H, *J* = 8 Hz), 7.65 (s, 1H), 7.01 (d, 2H, *J* = 8 Hz), 3.91 (s, 3H); 7d: ^1^H NMR (400 MHz; CDCl_3_) *δ* (ppm): 7.96 (m, 1H), 7.89 (m, 1H), 7.76 (m, 1H), 7.74 (m, 1H), 7.43 (t, 1H); 7e: ^1^H NMR (400 MHz; CDCl_3_) *δ* (ppm): 7.77 (d, 2H, *J* = 8 Hz), 7.72 (s, 1H), 7.69 (d, 2H, *J* = 8Hz); 7f: ^1^H NMR (400 MHz; CDCl_3_) *δ* (ppm): 7.87 (d, 2H, *J* = 8 Hz), 7.64 (s, 1H), 6.97 (d, 2H, *J* = 8Hz); 7g: ^1^H NMR (400 MHz; CDCl_3_) *δ* (ppm): 8.05 (s, 4H), 7.26 (s, 2H); 7h: ^1^H NMR (400 MHz; CDCl_3_) *δ* (ppm): 8.3 (s, 1H), 8.08 (m, 1H), 7.93 (m, 4H), 7.65 (m, 2H); 7i: ^1^H NMR (400 MHz; CDCl_3_) *δ* (ppm): 7.37 (s, 1H), 7.26 (s, 1H), 6.36 (d, 1H), 2.47 (s, 3H); 7j: ^1^H NMR (400 MHz; CDCl_3_) *δ* (ppm): 9.52 (s, 1H), 9.0 (d, 2H, *J* = 8 Hz), 8.71 (s, 1H), 8.07 (s, 2H), 7.74 (m,4H).

## Results and discussion

### Synthesis and characterization of ligand L and cage CA1

**Scheme 1:**
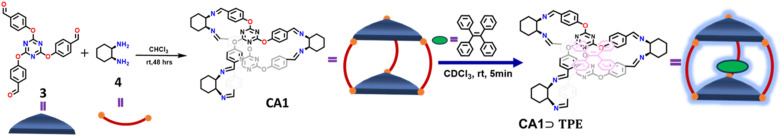
Reaction scheme for the synthesis of cage CA1 and CA1⊃TPE.

Triazine-based *tri*-aldehyde ligand L was synthesized by a substitution reaction between cyanuric chloride (1) and 4-hydroxybenzaldehyde (2) in an acetone-water solvent mixture under basic conditions at room temperature in 96% yield (Scheme S1). The synthesized ligand (L) was characterized by ^1^H and ^13^C NMR spectroscopy as well as mass spectrometry ESI-MS (Fig. S1, S2 and S6). The shape-persistent triazine-based organic-imine cage CA1 was synthesized using the DCC approach by the Schiff base condensation reaction of *tri*-aldehyde ligand L with *cis-*cyclohexane-1,2-diamine in a 2 : 3 molar ratio in chloroform, resulting in the formation of a three-dimensional cage structure (Scheme 1).

**Fig. 1:**
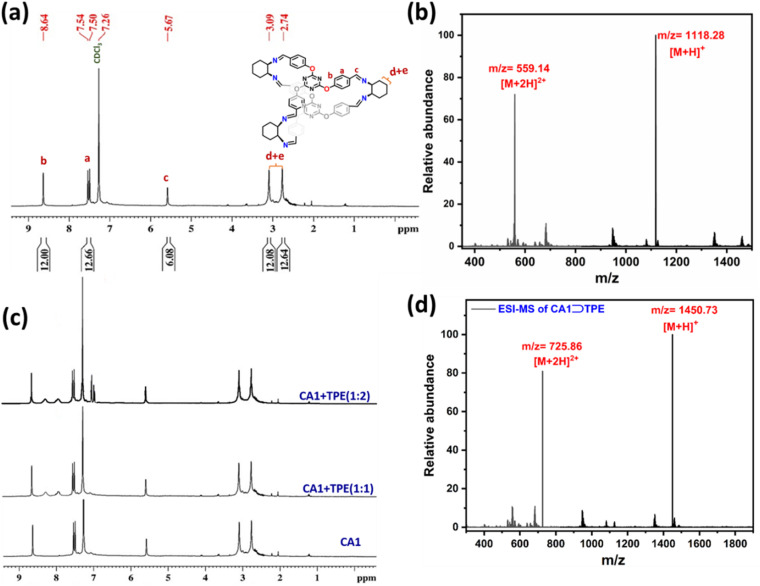
(a) ^1^H NMR (CDCl_3_ and 400 MHz) of CA1, (b) ESI-MS of CA1 in MeOH, (c) ^1^H NMR (CDCl_3_, 400 MHz) titration of CA1 with TPE, and (d) ESI-MS of CA1⊃TPE in MeOH.

The formation of the cage compound was first investigated by ^1^H NMR, indicating the absence of an aldehydic proton at 9.99 ppm in the cage complex of CA1 compared to L. The subsequent appearance of the CH_2_ proton at 5.67 ppm confirms the complete consumption of the aldehyde upon reaction with amine derivatives (Fig. 1a). Moreover, the two-dimensional diffusion-ordered ^1^H NMR spectrum (DOSY) of cage CA1 exhibits a single diffusion coefficient at −log *D* = 9.38, which confirms the formation of a single species of cage complex CA1 (Fig. S3). The stoichiometric compositions of CA1 were investigated through ESI-MS spectrometric analysis, giving peaks at *m*/*z* = 1118.28 and 559.14 corresponding to [M + H]^+^ and [M + 2H]^2^, respectively, which revealed that 2 equivalents of *tri*-aldehyde and 3 equivalents of diamine condensed to form a [3 + 2] molecular cage (Fig. 1b).

### Host-guest interaction of CA1 and tetraphenylethene (TPE)

The designed organic cage, CA1, includes an internal cavity made up of a triazine panel characterized by a significant electron deficiency, thereby facilitating the possibility of guest encapsulation *via* host–guest interactions. Therefore, we selected an aggregation-induced emissive (AIE) propeller shape molecule, tetraphenylethene (TPE), possessing characteristic emissive properties as a guest molecule for encapsulation within the cage cavity of CA1. The successful encapsulation of TPE within the cavity of CA1 in a 1 : 1 molar ratio was achieved by host–guest interaction, which was first investigated by ^1^H NMR titration of TPE, with increasing molar ratio of TPE in CA1 solution in CDCl_3_ resulting in significant chemical shift changes, as observed in the proton signals of both the cage and the guest (Fig. 1c). Upon mixing the host cage CA1 with TPE guest in a 1 : 1 molar ratio, a set of new peaks appeared at 8.26 and 7.96, corresponding to the encapsulated TPE aromatic proton, which is slightly upfield shifted compared to free TPE in CDCl_3_ (Fig. S4). This upfield shift corresponding to the aromatic proton of encapsulated TPE is due to the shielding effects caused by the electron-deficient cage cavity. These spectral changes confirm the quantitative encapsulation of TPE inside the cage, wherein TPE is situated in the internal cavity of the cage, experiencing anisotropic shielding from the triazine and aryl walls. Further increasing the molar ratio of TPE (1 : 2) gave a free TPE proton peak, suggesting effective 1 : 1 host–guest encapsulation (Fig. 1c). The encapsulation of TPE was also supported by DOSY ^1^H NMR, which showed a single band of diffusion coefficient at −log *D* = 9.74 corresponding to the encapsulated aromatic proton of TPE and cage complex CA1 (Fig. S5). The formation of a 1 : 1 host–guest complex of CA1⊃TPE was also established from ESI-MS, which gave peaks at *m*/*z* = 1450.73 and 725.86 corresponding to [M + H]^+^ and [M + 2H]^2+^, respectively (Fig. 1d).

### Photophysical properties

The photophysical behaviours of ligand L and cage CA1 and CA1⊃TPE were systematically investigated by UV-visible and fluorescence spectroscopy in THF solution. The UV-visible spectra of *tri*-aldehyde L exhibit a single absorption band at 205 nm, which is attributed to the n–π* electronic transitions localized within the aldehyde-substituted triazine aromatic system. CA1 exhibited a prominent absorption band at 262 nm attributed to n–π* transitions localized within the aromatic framework of the triazine and amine moieties (Fig. S10). The formation of a 1 : 1 host–guest complex of CA1⊃TPE was also established from UV-visible titration studies of TPE in cage solution by Job's plot (Fig. S11). The encapsulation of the electron-rich AIE-active TPE molecule with the electron-deficient cage cavity causes a slight blue shift of 2 nm in the absorbance of CA1, indicating the retention of n–π* transition character of CA1, while encapsulated TPE exhibits absorbance at 320 nm, which is attributed to π–π* transition of its aromatic backbone (Fig. S10). The emission intensity of CA1⊃TPE was also investigated in different solvents with low to high polarity. Increasing the solvent polarity from hexane {*λ*_em_ (CA1⊃TPE) = 405 nm} to DMSO {*λ*_em_ (CA1⊃TPE) = 440 nm} leads to a bathochromic shift of emission intensity of about 35 nm due to the stabilization of n–π* transitions in a polar solvent (Fig. S1 2 and Table S1).

### AIE behaviour of the CA1⊃TPE

**Fig. 2:**
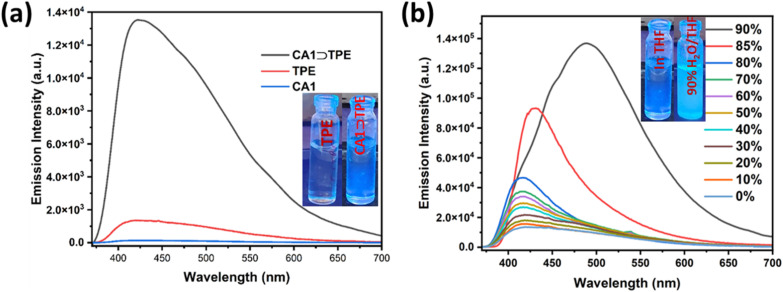
Fluorescence emission spectra of (a) CA1, TPE and CA1⊃TPE in THF (*c* = 10^−5^ M for each), and (b) CA1⊃TPE with increasing water fraction in THF (*λ*_ex_ = 320 nm and *c* = 10^−5^ M CA1⊃TPE).

CA1 is nearly non-emissive in THF, while TPE is weakly emissive at 419 nm in the THF solution. However, host–guest complex CA1⊃TPE displays blue emission at 424 nm with 10-fold enhanced emission intensity (Fig. 2a). The TPE is an AIE active molecule, which is weakly emissive in dilute solution but becomes highly emissive due to restrictive rotation of its phenyl ring in the aggregate state, which activates its radiative decay pathways.

Therefore, encapsulation of TPE within the cage framework partially freezes its phenyl ring rotation due to cage constraint, which enhances its emission intensity in line with aggregation-induced conformational rigidity. A gradual increase in water fraction in THF, which is a poor solvent for host–guest complex CA1⊃TPE causes aggregate formation, which further boosts its emission intensity with a large Stokes shift due to the pronounced AIE effect. Maximum emission enhancement was achieved in a 90% water/THF mixture, where the CA1⊃TPE complex exhibited a sharp increase in its emission intensity at 488 nm with a 64 nm red shift upon excitation at 320 nm (Fig. 2b). This phenomenon is attributed to the further restriction of the intramolecular rotation of the TPE units within the confined and aggregated environment of the cage assembly. The aggregation process effectively suppresses non-radiative decay pathways, resulting in enhanced radiative transitions. Compared to the dilute solution state, the fluorescence intensity in the aggregated state increased by a factor of 10.12-fold, highlighting the efficient AIE activity of the CA1⊃TPE system. Visual inspection under UV light further supports the formation of aggregates at higher water content as the blue emission intensity progressively increases with increasing water fraction. The observed AIE behaviour of CA1⊃TPE confirms the ability of the triazine-based cage to act as an efficient fluorescent scaffold by spatially confining TPE units and promoting emissive states in aggregated or encapsulated forms.

### Morphological characterization of the aggregate of CA1⊃TPE

**Fig. 3:**
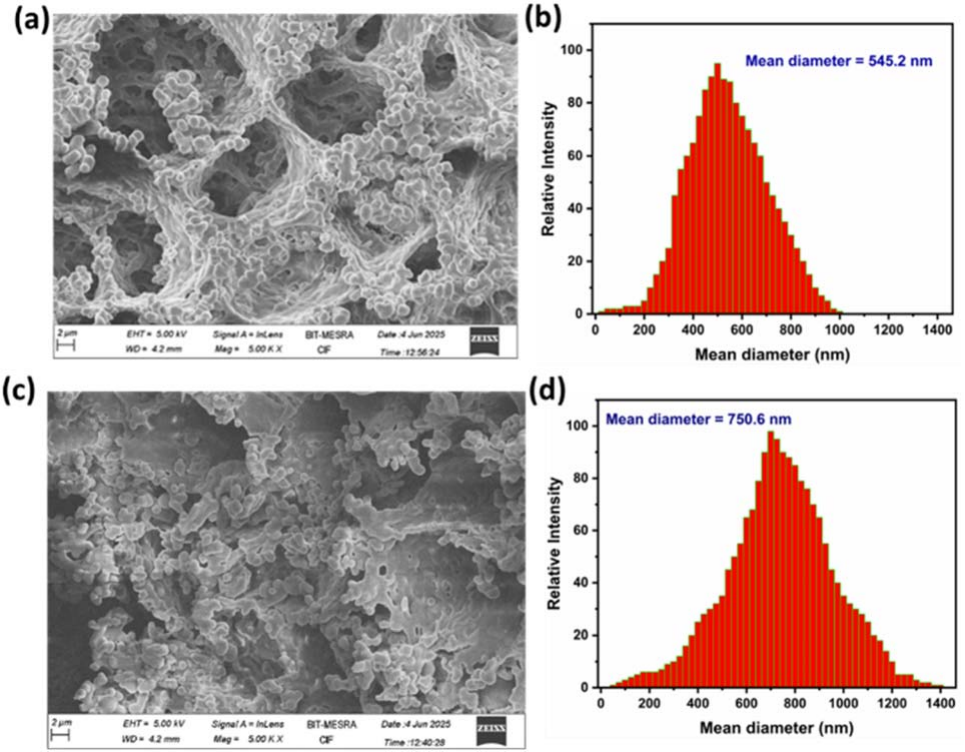
(a) FESEM image of CA1⊃TPE in 90% H_2_O/THF, (b) DLS data for size distribution pattern of CA1⊃TPE in 90% H_2_O/THF, (c) FESEM image of CA1⊃TPE@RhB in 90% H_2_O/THF, and (d) DLS data for the size distribution pattern of CA1⊃TPE@RhB in 90% H_2_O/THF.

The CA1⊃TPE shows the highest emission in a mixture of 90% water/THF due to the formation of aggregates; thus, the morphological properties of these aggregates were examined through field emission scanning electron microscopy (FESEM). For the morphological characterization of CA1⊃TPE, we prepared an equimolar ratio of CA1 and TPE by dissolving them in THF to create a stock solution of 10^−3^M in THF. Subsequently, in a separate 5 mL vial, we added 30 μL of the 10^−3^ M CA1⊃TPE solution in THF, along with 270 μL of THF and 2700 μL of water, resulting in a total of 3 mL of a 10^−5^ M CA1⊃TPE solution in a solvent mixture of 90% water/THF (v/v). We recorded the DLS for 3 mL of 10^−5^ M CA1⊃TPE in a 90% water/THF mixture (Fig. 3b). For FESEM analysis, 100 μL of 10^−5^M solution of CA1 and CA1⊃TPE in 90% water/THF mixture were separately drop cast on a glass wafer, followed by vacuum drying. The FESEM analysis reveals the formation of nano-aggregates of spherical morphologies typically ranging in diameter from 200 to 350 nm for CA1 and 450 to 600 nm for CA1⊃TPE, suggesting a uniform and compact packing of the CA1 and CA1⊃TPE system upon aggregation (Fig. S18, S19 and 3a). The aggregates appeared as dense, non-crystalline spherical assemblies, which are characteristics of AIE-active supramolecular systems formed through hydrophobic interactions and π–π stacking in aqueous-rich environments. DLS measurements of the CA1 and CA1⊃TPE system in 90% water/THF medium further supported the formation of aggregates with a mean hydrodynamic diameter of 292.8 nm for CA1 and 545.2 nm for CA1⊃TPE, which is consistent with the size distribution observed in FESEM (Fig. S20 and 3b). These morphological observations provide compelling evidence for the formation of discrete supramolecular nano-assemblies in aqueous environments, driven by the aggregation of the cage and host–guest complexes. The spherical architecture and nanoscale dimensions of the aggregates are advantageous for light-harvesting and photocatalytic applications, as they facilitate efficient energy transfer, reduce exciton quenching, and enhance emission due to restricted molecular motion in the confined environment. Overall, the morphological characterization corroborates the photophysical data, confirming the successful formation of emissive nanoaggregates through aggregation-induced self-assembly of the TPE-encapsulated cage in water-rich media.

### Artificial light harvesting function of CA1⊃TPE

**Fig. 4:**
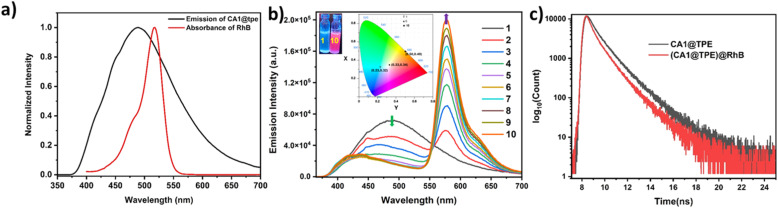
(a) Normalised plots of the emission of CA1⊃TPE and the absorbance of RhB. (b) Fluorescence titration spectra of CA1⊃TPE (10^−5^ M in 90% H_2_O/THF and *λ*_ex_ = 320 nm) with a gradual addition of RhB (in 90% H_2_O/THF) from 10^−7^ M to max. 10^−6^ M. (Inset) Photograph of CA1⊃TPE in 90% H_2_O/THF mixture (left); CA1⊃TPE + RhB in 90% H_2_O/THF mixture (right) under UV-light@ 320 nm (inset). The chromaticity coordinate changes (1931 CIE plot) as CA1⊃TPE (10^−5^ M, 90% H_2_O/THF mixture, and *λ*_ex_ = 320 nm) titrated against RhB in 90% H_2_O/THF mixture from 10^−7^ M to max. 10^−6^ M. (c) Fluorescence decay profile for CA1⊃TPE (black, *λ*_ex_ = 320 nm, and *c* = 10^−5^ M) and CA1⊃TPE + RhB (red, *λ*_ex_ = 320 nm, and 10^−5^ M [CA1⊃TPE] + 10^−6^ M [RhB]) in 90% water/THF.

Host–guest complex CA1⊃TPE is blue emissive in the aggregate state, and its maximum intensity was observed in a 90% water/THF mixture. Therefore, we explored the light-harvesting function through the FRET process of CA1⊃TPE in a 90% water/THF mixture in the aggregate state, as the aggregate did not exhibit any emission self-quenching over time due to precipitation. We investigated the energy transfer behaviour of CA1⊃TPE *via* the FRET process, where it acted as an energy donor to the water-soluble fluorescent dye RhB, which acted as an energy acceptor molecule. The selection of RhB is fortunate because its absorbance shows considerable overlap with the emission of energy donor molecule CA1⊃TPE, which is a preliminary requirement for the FRET process (Fig. 4a). The energy transfer process between CA1⊃TPE (in 90% water/THF) to RhB (in 90% water/THF) was established by fluorescence titration experiments with the gradual addition of RhB (10^−5^M) to a fixed concentration of the CA1⊃TPE (*c* = 10^−5^M). When 30 μL of 10^−5^ M solution of RhB was gradually added to the CA1⊃TPE solution, a sharp decrease in emission intensity of CA1⊃TPE at 488 nm with a slight blue shift was observed, while emission intensity at 576 nm corresponding to RhB increased upon donor excitation at 320 nm. The increase in the emission intensity of RhB, accompanied by a decrease in the emission intensity of RhB, suggests the energy transfer from donor CA1⊃TPE to acceptor RhB. When 300 μL 10^−5^M solution of RhB was added 10^−5^M solution of donor, the energy transfer process was saturated, corresponding to a donor : acceptor ratio of 10 : 1 (Fig. 4b and S13a). Control experiments with either RhB or cage-TPE alone confirmed that the observed enhanced emission was specifically due to energy transfer. When 300 μL of a 10^−5^ M RhB solution was introduced to 3 mL of a 90% water/THF mixture in the absence of CA1⊃TPE, it was found to be non-emissive upon excitation at 320 nm, which clearly rules out the emission by direct excitation of RhB and supports the FRET process between CA1⊃TPE and dye RhB (Fig. S15). In the process of FRET, a visual colour change was noticed from emissive blue (for CA1⊃TPE) to emissive pink (CA1⊃TPE@RhB) under a UV lamp of 320 nm. Fluorescence colour change was also monitored by the CIE chromaticity diagram, where the blue solution of CA1⊃TPE exhibits CIE coordinate (0.23 : 0.32), which changes to the pink solution of CA1⊃TPE@RhB with CIE coordinate (0.50 : 0.49) (Fig. S13b). Ground state interaction between the energy donor and energy acceptor was checked by UV-visible titration in 90% water/THF by the gradual addition of 30 μL of 10^−5^ M solution of RhB to CA1⊃TPE (*c* = 10^−5^M) solution. UV-visible titration studies show an increase in absorbance of RhB at 518 nm, while the absorbance of CA1⊃TPE at 320 nm remains almost constant, clearly ruling out the existence of any ground state interaction between CA1⊃TPE and RhB (Fig. S14). The morphological characterization of the FRET pair (CA1⊃TPE + RhB) in the aggregate state was monitored by FESEM analysis, suggesting the formation of spherical nanoaggregates, while the mean average diameter of the aggregate assembly was 750.6 nm according to DLS measurements (Fig. 3c and d). The existence of well-formed spherical nano-aggregates of CA1⊃TPE + RhB systems confirms the energy transfer process in the aggregate state. The FRET process between CA1⊃TPE and RhB was supported by Time-Correlated Single-Photon Counting (TCSPC) measurements. The TCSPC measurements showed a significant decrease in the fluorescence lifetime of the donor upon RhB addition. The average lifetime of the CA1⊃TPE donor was 2.58 ns, which decreased to 2.14 ns with the addition of RhB at a 10 : 1 donor-to-acceptor ratio (Fig. 4c, Table S2, and (eqn (1)). The FRET process between CA1⊃TPE and RhB was further supported by fluorescence quantum yield measurement (*Φ*_F_), where CA1⊃TPE (*c* = 10^−5^ M) exhibits *Φ*_F_ = 13.49, which increased to *Φ*_F_ = 16.18 upon formation of the FRET pair of CA1⊃TPE@RhB (Fig. S16 and S17). The maximum energy transfer efficiency (*Φ*_ET_) of CA1⊃TPE to RhB was calculated (eqn (2)) as 88.9% at a donor/acceptor ratio of 10 : 1 in a 90% H_2_O/THF solution (Table S3). In another experiment, we measured the emission intensity FRET pair (CA1⊃TPE@RhB) and donor (CA1⊃TPE) at donor excitation (*λ*_ex_ = 320 nm) and the emission intensity of the FRET pair at acceptor excitation (*λ*_ex_ = 520 nm), which gave an antenna effect of 11.1 (Fig. S24, eqn (3), and Table S4). These results clearly demonstrate that the AIE-active CA1⊃TPE forms an efficient FRET pair system and exhibits an efficient artificial-light harvesting system (LHS).

### Photocatalytic activity

To evaluate the functional applicability of the artificial LHS CA1⊃TPE@RhB, we explored its photocatalytic performance in the Knoevenagel condensation reaction. For photocatalytic activity, we explored the condensation reaction between benzaldehyde (5a) and malononitrile (6) in an aqueous medium. The photocatalytic reaction was optimized using 7.5 mol% CA1⊃TPE and 1.5 mol% RhB catalyst at a temperature of 50 °C in open air under visible light, resulting in the desired benzylidene malononitrile product with an isolated yield of 97% after 1 hour (Table 1). In contrast, reactions carried out using RhB alone under identical conditions resulted in only a 25% yield, while the CA1⊃TPE complex alone showed a trace amount of product formation. Furthermore, in the absence of visible light, CA1⊃TPE@RhB does not demonstrate any catalytic effectiveness, which results in only trace amounts of product formation, thereby confirming the necessity of visible light for photocatalytic regulation.

**Table 1:** Optimisation of reaction conditions for the Knoevenagel condensation reaction^a^

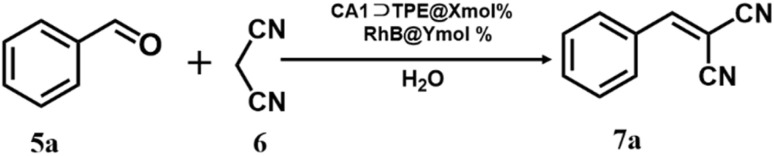							
Entry	CA1⊃TPE (X mol%)	RhB (y mol%)	Time (in h)	Reaction temperature	Light source	In the presence of air	% yield 7a^b^
1	7.5	1.5	3	Rt	Visible	Yes	76
2	7.5	1.5	1	Rt	Visible	Yes	46
3	7.5	1.5	1	50 °C	Visible	Yes	97
4	7.5	1.5	1.5	50 °C	Visible	Yes	96
5	7.5	1	1	50 °C	Visible	Yes	78
6	7.5	0.5	1	50 °C	Visible	Yes	54
7	7.5	0	1	50 °C	Visible	Yes	Trace
8	5	1.5	1	50 °C	Dark	Yes	Trace
9	0	1.5	1	50 °C	Visible	Yes	Trace
10	7.5	1.5	1	50 °C	Visible	No^c^	10
11^d^	7.5	1.5	1	50 °C	Visible	Yes	Trace

a Reaction conditions: benzaldehyde 5a (0.97 mmol), malononitrile 6 (1.95 mmol), water (2 ml), stirring.

b Crude yields isolated by extraction with ethyl acetate and water. A Philips 50 W LED bulb (5000 Lm, 7500 K) is used as a visible light source.

c Reaction in the absence of air was performed under an inert atmosphere (in the presence of nitrogen).

d Photocatalytic reaction in presence of radical scavenger TEMPO (0.97 mmol).

**Scheme 2:**
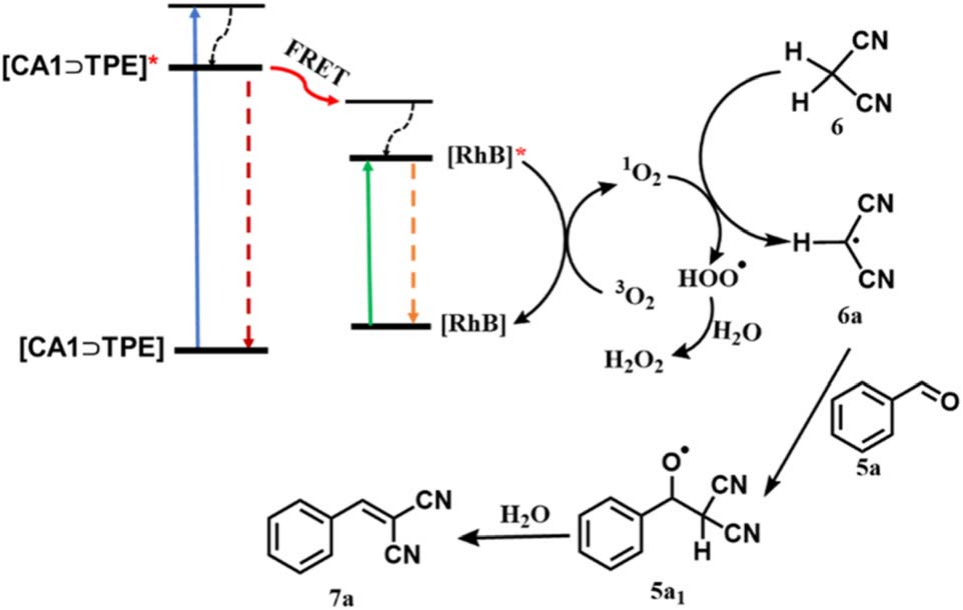
Proposed reaction pathways of photocatalyzed reaction mechanism for Knoevenagel condensation of benzaldehyde (5a) and malononitrile (6).

The photocatalytic reaction proceeds through the photosensitization of RhB by a FRET process, where CA1⊃TPE transfers its excited energy to RhB (Scheme 2). The photoexcited [RhB]* retransfers its excited state energy to ^3^O_2_ and generates ^1^O_2_ (singlet oxygen). Formation of singlet oxygen during the course of the reaction was confirmed by fluorescence titration of anthracene (in THF) with the reaction mixture at different time intervals (Fig. S36). When anthracene was titrated with the reaction mixture from *t* = 0 min to *t* = 60 min, its emission intensity decreased as the formed ^1^O_2_ during the reaction was captured by anthracene (Fig. S36). The ^1^O_2_ subsequently captures a proton from the 6 to give malononitrile radical intermediate (6a) and H_2_O_2_. In the next step, 6a reacted with benzaldehyde (5a), generating radical intermediate 5a_1_, which was subsequently quenched with water to produce product benzylidene malononitrile 7a. In the controlled experiment, when the reaction was performed with the radical scavenger TEMPO, a trace amount of product formation was observed, suggesting the operation of a catalytic cycle by radical pathways (Table 1 and Entry 11). The formation of radical intermediate 6a was captured by TEMPO and confirmed by ESI-MS of the 6a-TEMPO adduct (Fig. S37). Additionally, the formation of H_2_O_2_ in the reaction was confirmed by the starch-KI solution (Table S5).

The scope of the photocatalyst CA1⊃TPE + RhB was further examined using a range of substituted benzaldehydes with electron donating substituents (*i.e.,* −4-CH_3_-Ph (5b), −4-OCH_3_-Ph (5c), −4-OH-Ph (5f), and -2-napthyl (5h)) and electron-withdrawing group substituents (*i.e.*, −3-Br-Ph (5d) and −4-Br-Ph (5e)); the reaction gave excellent product yields (92–98%) and was well characterized by ^1^H NMR (Table 2 and Fig. S25–S34). The LHS system was found to be an effective photocatalyst for highly insoluble anthracene-9-carboxaldehyde, with a good product yield of 77%. The photocatalyst CA1⊃TPE @RhB was recovered by extraction and used further for another cycle of catalysis reaction. The CA1⊃TPE@RhB exhibits no degradation while maintaining its catalytic efficiency for up to 5 cycles of catalytic reaction (Fig. S35). The improved catalytic activity of the CA1⊃TPE@RhB as an artificial LHS is attributed to the efficient energy relay from the AIE-active donor CA1⊃TPE to the RhB acceptor *via* the FRET mechanism, which subsequently facilitates the photocatalytic reaction. The broader absorption of the CA1⊃TPE donor in the UV-visible region and its efficient energy transfer to RhB extend the light-harvesting window and amplify the dye's photocatalytic activity. These results validate the efficacy of the nano-aggregate of CA1⊃TPE@RhB as an efficient light-harvester and act as a visible-light-driven photocatalyst under mild aqueous conditions. The CA1⊃TPE in the aggregate state mimics natural photosynthetic antenna behaviour by channelling excitation energy toward an acceptor dye, which in turn initiates a catalytic condensation process.

**Table 2:** Catalysis of the Knoevenagel condensation reaction by artificial LHSs (CA1⊃TPE@RhB)^a^

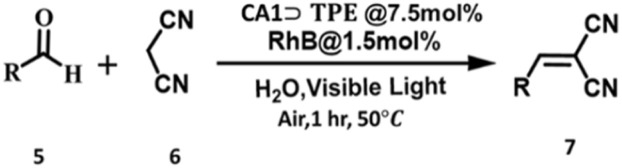			
Entry	R	Product	% yield^b^
1	Ph (5a)	7a	97
2	4-CH_3_-Ph (5b)	7b	92
3	4-OCH_3_-Ph (5c)	7c	96
4	3-Br-Ph (5d)	7d	96
5	4-Br-Ph (5e)	7e	92
6	4-OH-Ph (5f)	7f	96
7	Terephthyl (5g)	7g	37
8	2-Napthyl (5h)	7h	98
9	Furfural (5i)	7i	88
10	9-Anthryl (5j)	7j	77

a Reaction conditions: benzaldehyde 5a–j (0.97 mmol), malononitrile 6 (1.95 mmol), water (2 ml), CA1⊃TPE (7.5 mol%), RhB (1.5 mol%), stirring at 50 °C temperature in the open air.

b Crude yields isolated by extraction with ethyl acetate and water. A Philips 50 W LED bulb (5000 Lm, 7500 K) is used as a visible light source.

## Conclusions

In summary, we reported a facile synthesis of a triazine-based supramolecular organic-imine cage using the DCC approach. The presence of an electron-withdrawing group of the triazine core promotes the encapsulation of the AIE active TPE molecule, resulting in the formation of a 1 : 1 host–guest complex. The rigid cage framework's encapsulation of TPE effectively suppresses non-radiative decay, resulting in a bright blue emission that was further intensified in a 90% H_2_O/THF mixture owing to the formation of aggregates. The formation of the nano-aggregate of the cage-TPE system was systematically characterized by SEM and DLS measurements. In the aggregate state, the blue-emissive CA1⊃TPE serves as an energy donor to the organic dye Rhodamine B (RhB), which functions as an energy acceptor through the FRET process. This is well established by a decrease in lifetime, an increase in fluorescence quantum yield and a colour change from blue to pink. At a 10 : 1 donor–acceptor ratio (CA1⊃TPE: RhB), the FRET pair exhibits a maximum energy transfer efficiency of 89.9%, with a good antenna effect of 11. Furthermore, CA1⊃TPE@RhB acts as an efficient light-harvesting material for visible-light-driven photocatalytic condensation reaction between substituted benzaldehyde and malononitrile in green aqueous medium with enhanced yield compared to CA1⊃TPE and RhB alone. Overall, this work establishes a blueprint for the rational design of artificial photosynthetic systems with precise integration of AIE-active molecules by host–guest chemistry and serves as a versatile platform for visible-light-mediated photocatalysis in green solvents.

## Author contributions

C. K. executed the synthesis and characterization of the cage complexes, LHS systems, along with their photocatalytic applications. A. K. formulated the overall studies and contributed to the manuscript preparation. Both authors contributed to the discussion of the results and the writing of the manuscript.

## Conflicts of interest

The authors declare no conflicts of interest.

## Supplementary Material

RA-015-D5RA07378B-s001

## Data Availability

The data supporting this article have been uploaded as part of the supplementary information (SI). Supplementary information: ^1^H NMR, ^13^C NMR, DOSY NMR, ESI-MS, UV-visible, fluorescence, quantum yield, life-time measurement, SEM, and DLS data. See DOI: https://doi.org/10.1039/d5ra07378b.
