# Supplementary material for: Self-assembled [2 + 3] organic-imine cage as an artificial light harvester for the photocatalytic organic transformation in an aqueous medium
Source: RSC Adv. 2025 Nov 7;15(51):43366–76. doi: 10.1039/d5ra07378b (PMC12593196; doi:10.1039/d5ra07378b)
Supplement: RA-015-D5RA07378B-s001 [file RA-015-D5RA07378B-s001.pdf]

Supplementary Information (SI) for RSC Advances.
This journal is © The Royal Society of Chemistry 2025

Supporting Information

Self-assembled [2+3] organic-imine cage as an artificial light harvester for the photocatalytic organic transformation in aqueous medium

Atul Kumar,*[a] and Chanchala Kumari[a]

[a]Department of Chemistry, Birla Institute of Technology, Mesra, Ranchi-835215, India.
Email: atulkumar@bitmesra.ac.in

Reaction scheme

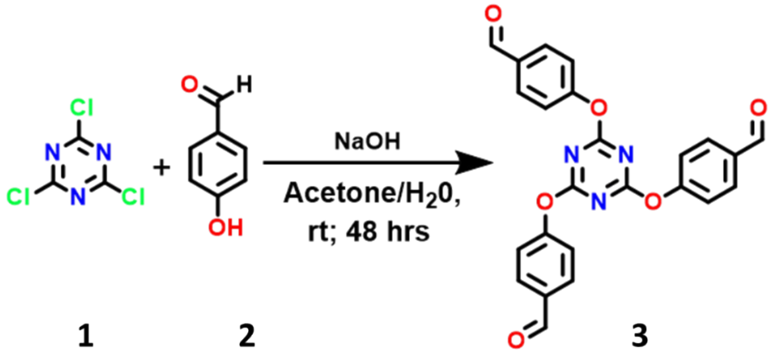

Scheme S1 Reaction scheme for the synthesis of ligand **L**.

Spectroscopic Characterization

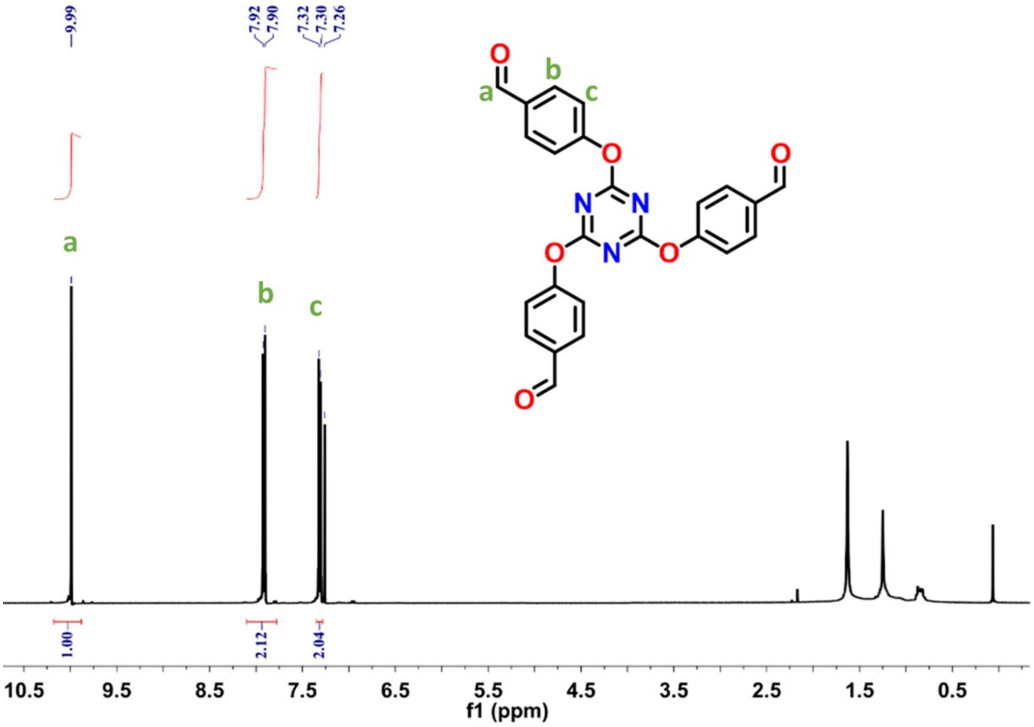

Fig. S1 ^{1}H NMR ($CDCl_3$, 400 MHz) of ligand **L**.

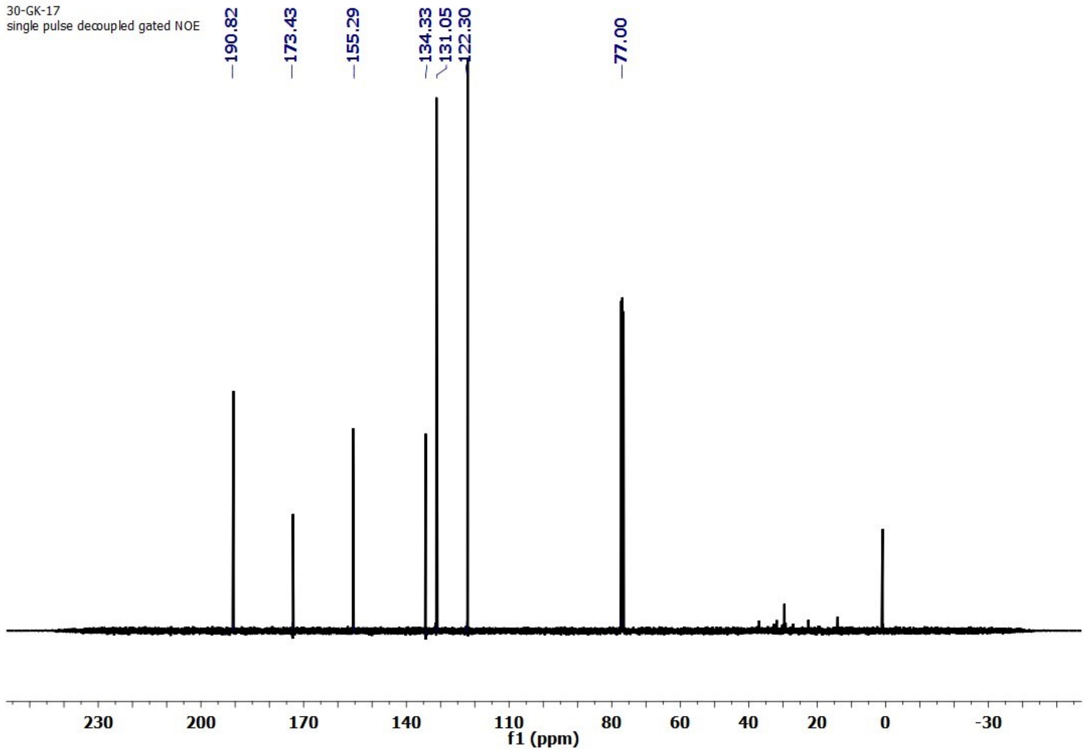

Fig. S2 ^{13}C NMR ($CDCl_3$, 400 MHz) of ligand **L**.

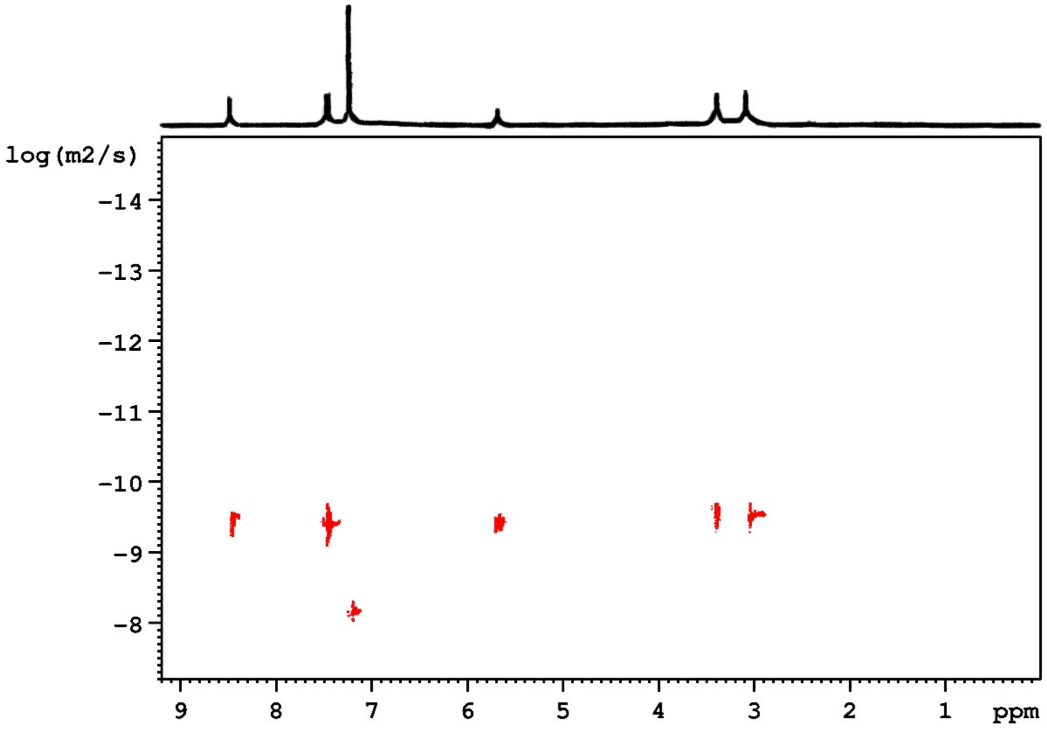

Fig. S3 ^{1}H DOSY NMR (400 MHz, $CDCl_3$) of **CA1**.

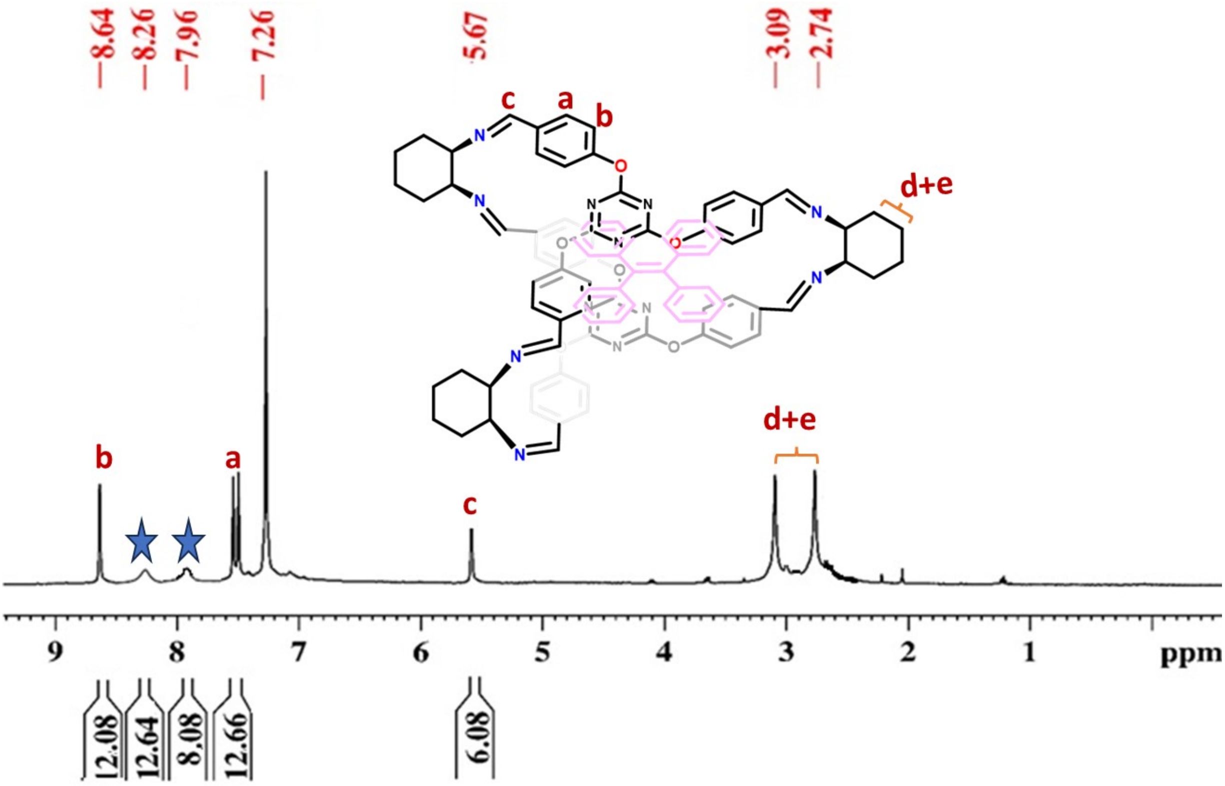

Fig. S4 ^{1}H NMR ($CDCl_3$, 400 MHz) of **CA1** $\supset TPE$ with 1:1 molar ratio of **CA1** and TPE.

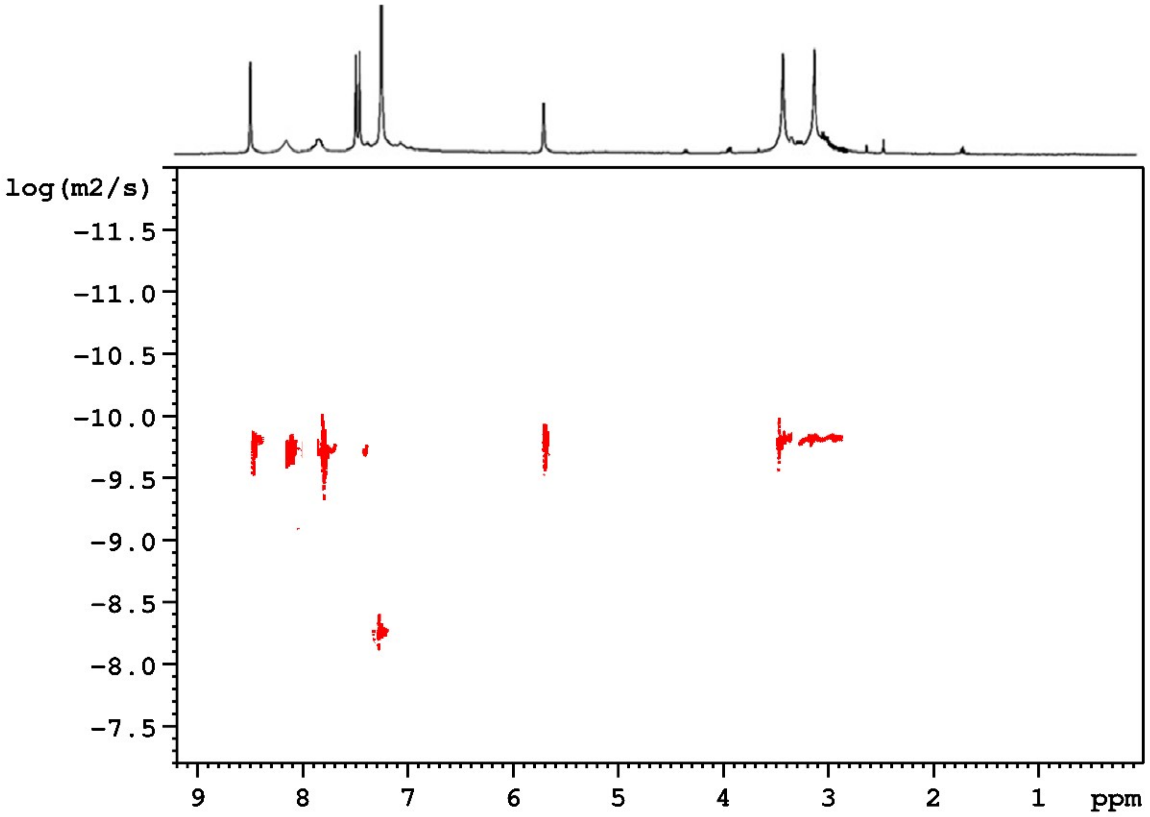

Fig. S5 DOSY ^{1}H NMR ($CDCl_3$, 400 MHz) of **CA1** $\supset TPE$ with 1:1 molar ratio of **CA1** and TPE.

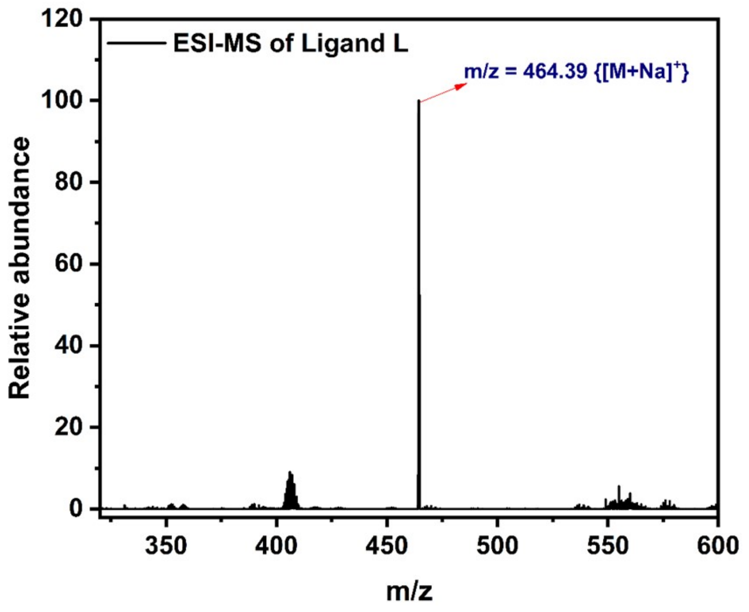

Fig. S6 ESI-MS spectrum of Ligand **L** in MeOH.

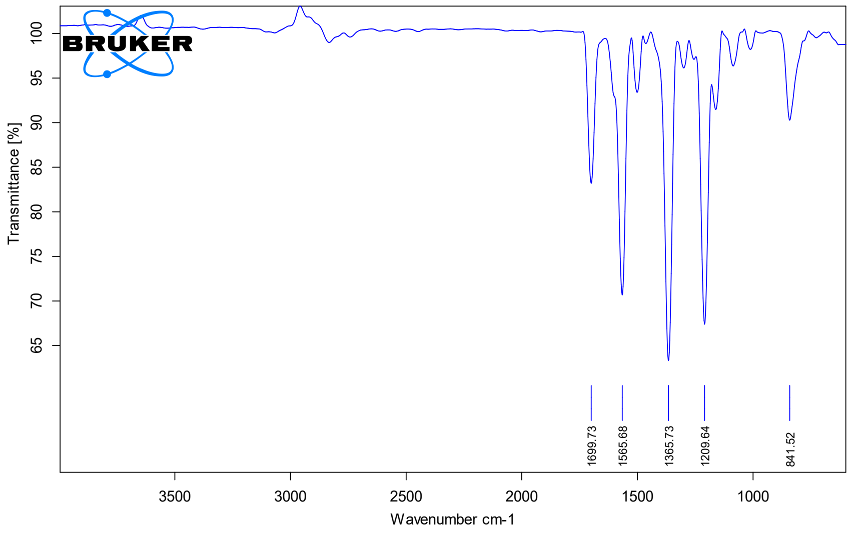

Fig. S7 FT-IR spectrum of **L**.

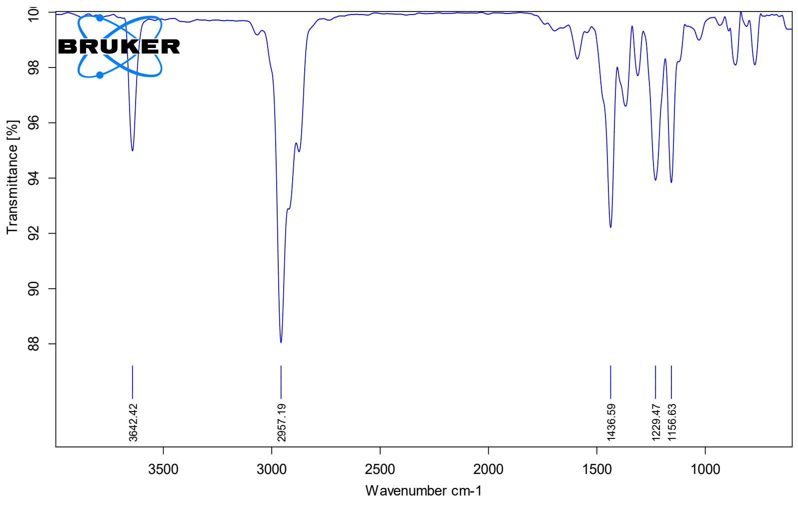

Fig. S8 FT-IR spectrum of **CA1**.

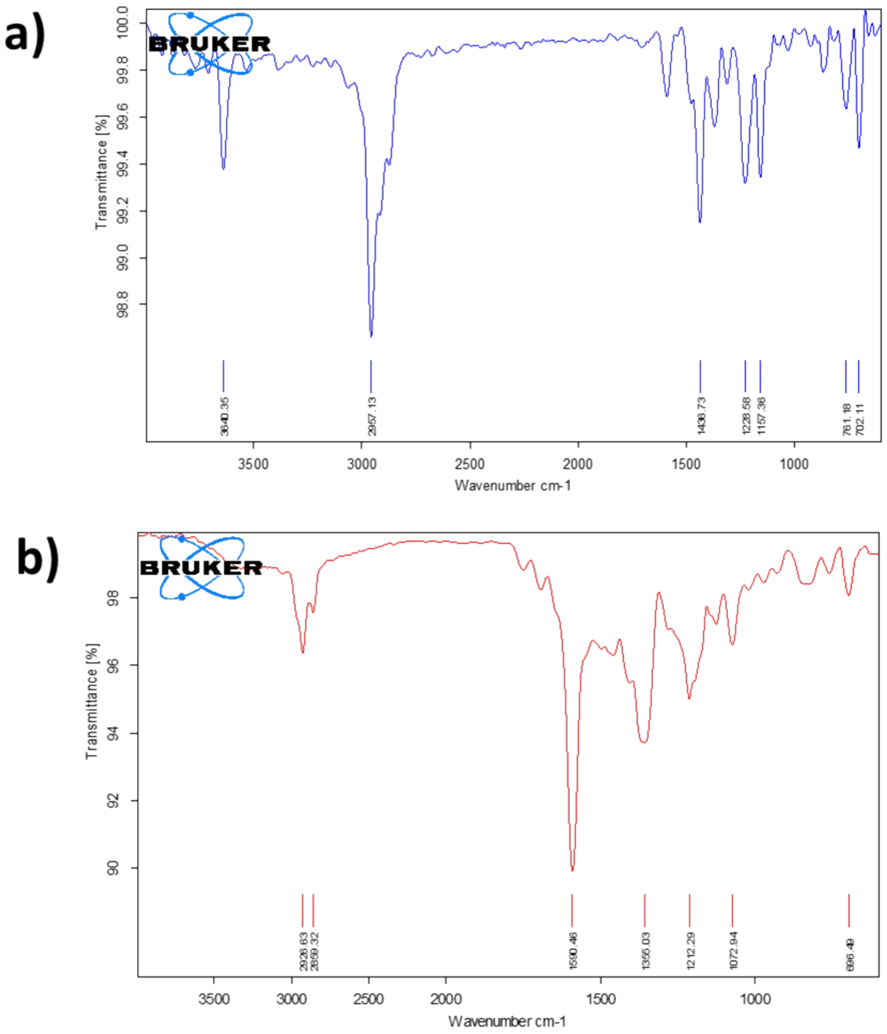

Fig. S9 FT-IR spectrum of a) **CA1** ⊃ TPE and b) photocatalyst **CA1** ⊃ TPE@RhB

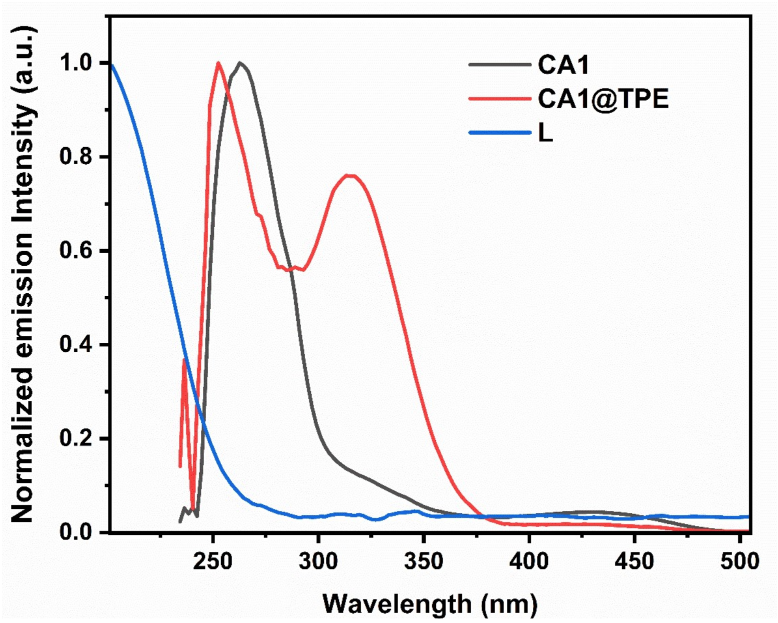

Fig. S10 UV-Visible spectra of **L**, **CA1** and **CA1** ⊃ TPE.

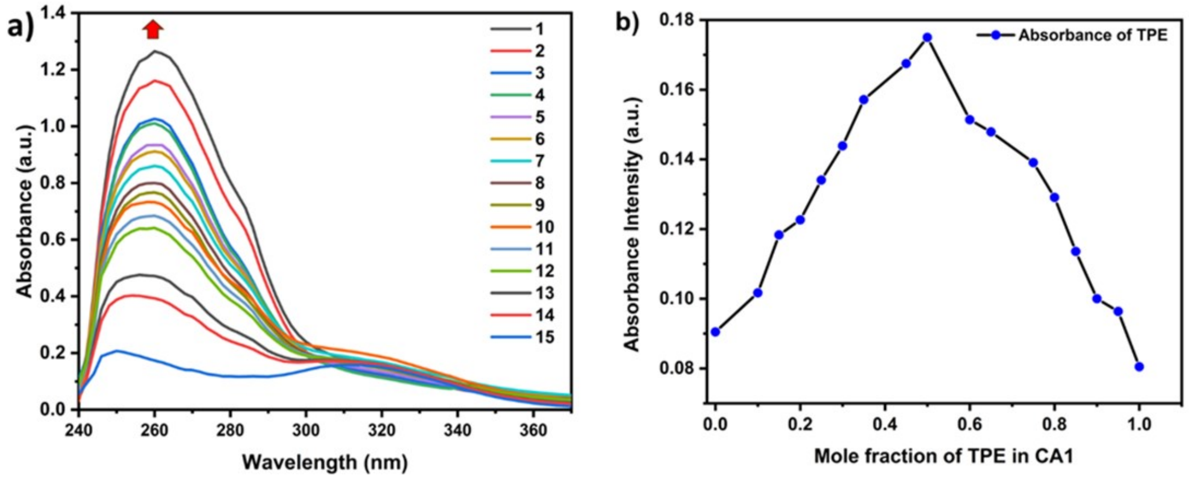

Fig. S11 a) UV-Vis spectra of **CA1** and TPE at different ratios in chloroform (0.1×10^{-5} M to 2×10^{-5} M TPE titrated against 10^{-5} M **CA1**); b) Job's Plot of UV-Vis titration spectra of **CA1** and TPE.

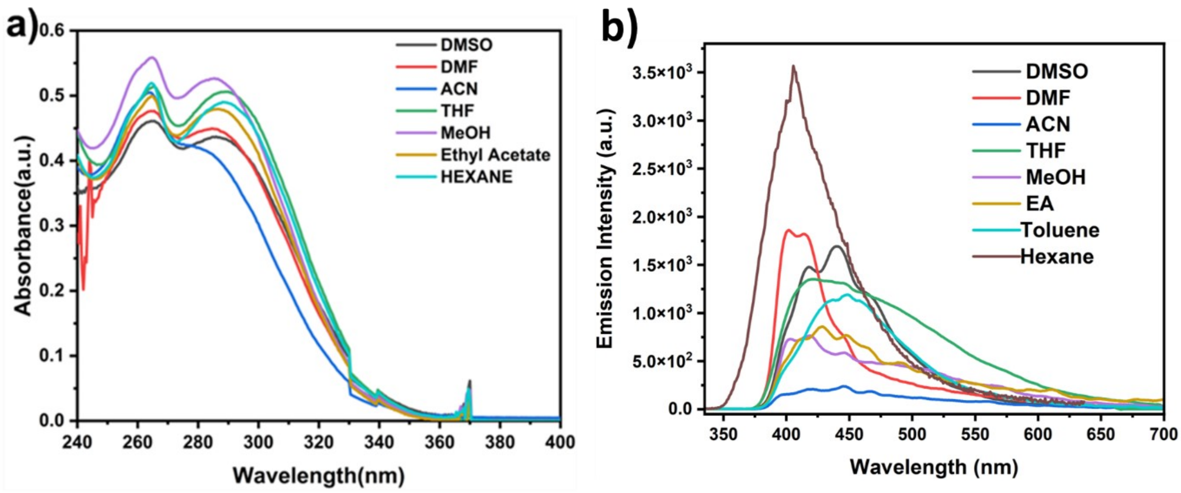

Fig. S12 a) UV-Vis spectra of **CA1** ⊃ TPE in different solvents [c = 10^{-5} M]; b) Fluorescence emission spectra of **CA1** ⊃ TPE in different solvents [$\lambda_{ex} = 300\ nm$, c = 10^{-5} M **CA1**].

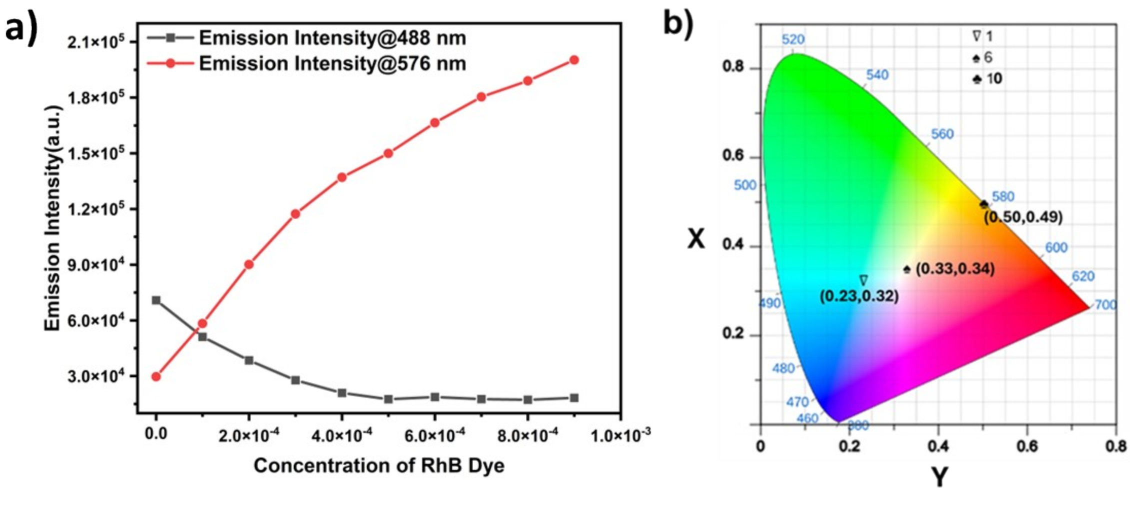

Fig. S13 a) Plot corresponding to fluorescence intensity change of **CA1** ⊃ TPE system at 485 and 578 nm. b) The 1931 CIE chromaticity coordinate changes as **CA1** ⊃ TPE(10^{-5} M, 90% H_2O/THF mixture, $\lambda_{ex} = 320\ nm$) titrated against RhB in 90% H_2O/THF mixture from 10^{-7} M to max. 10^{-6} M.

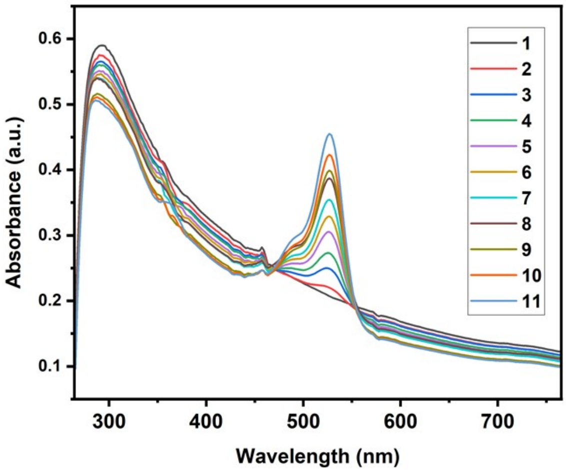

Fig. S14 UV-visible spectra of **CA1 ⊃ TPE** (10^{-5} M, 90% H_2O/THF mixture, λ_{max} = 320 nm) with gradual titration of RhB (90% water/THF) from 10^{-7} M to max. 10^{-6} M.

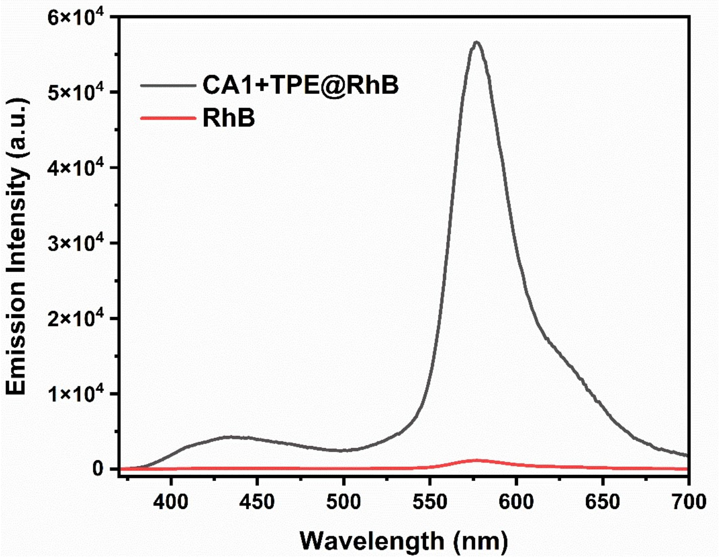

Fig. S15 Fluorescence emission spectra of **CA1** ⊃ TPE+RhB **(**10^{-5} M [**CA1** ⊃ TPE]+10^{-6} M [RhB], 90% H_2O/THF mixture, λ_{ex} = 320 nm) and RhB **(**10^{-6} M [RhB] in 90% H_2O/THF mixture, λ_{ex} = 320 nm)**.**

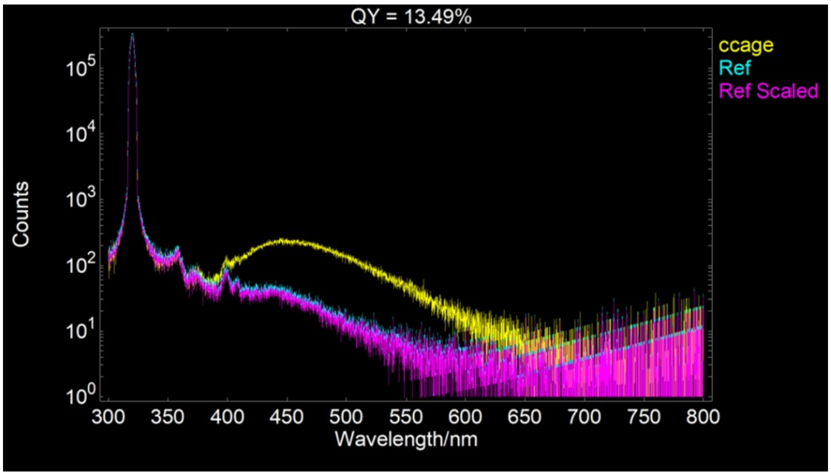

Fig. S16 Absolute fluorescence quantum yield of **CA1** ⊃ TPE in 90% water/THF. (c = 10^{-5} M [**CA1** ⊃ TPE]).

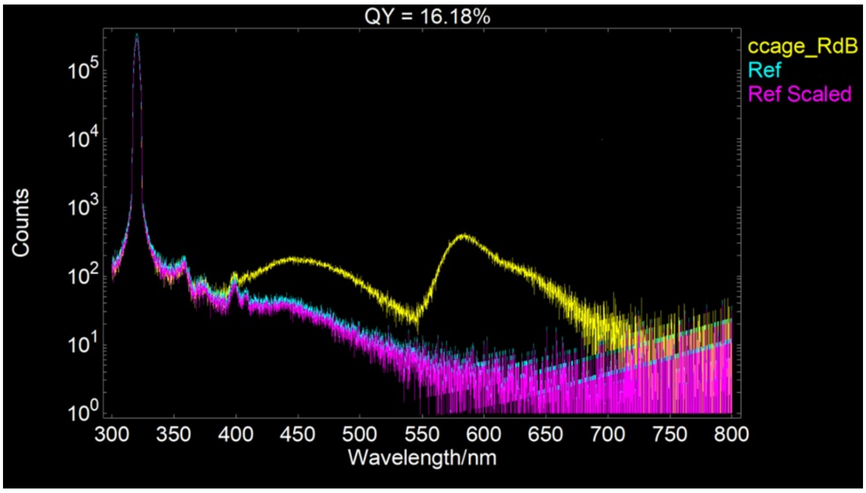

Fig. S17 Absolute fluorescence quantum yield of (**CA1** ⊃ **TPE**)@RhB in 90% water/THF. (c = 10^{-5} M [**CA1** ⊃ TPE] and c =10^{-6} M [RhB]).

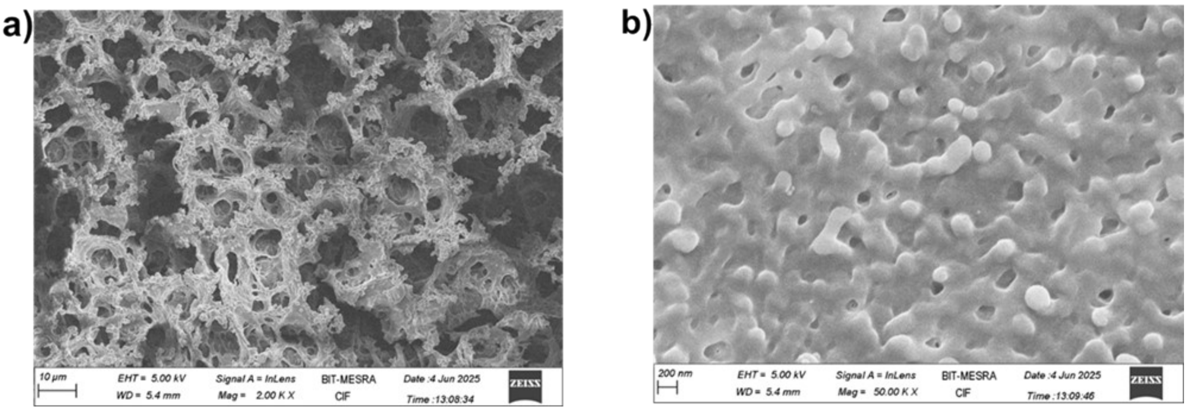

Fig. S18 FESEM image of **CA1**.

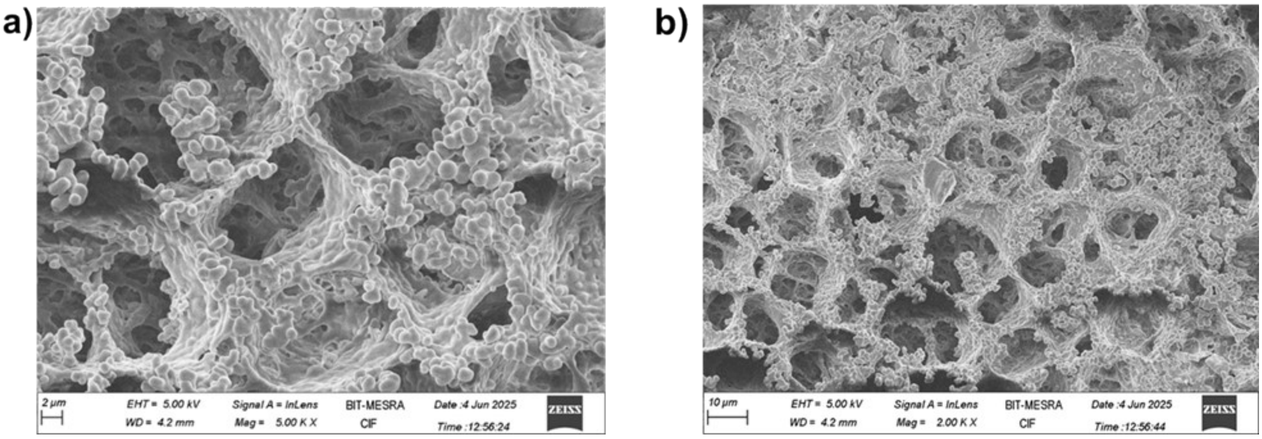

Fig. S19 FESEM image of **CA1** ⊃ TPE.

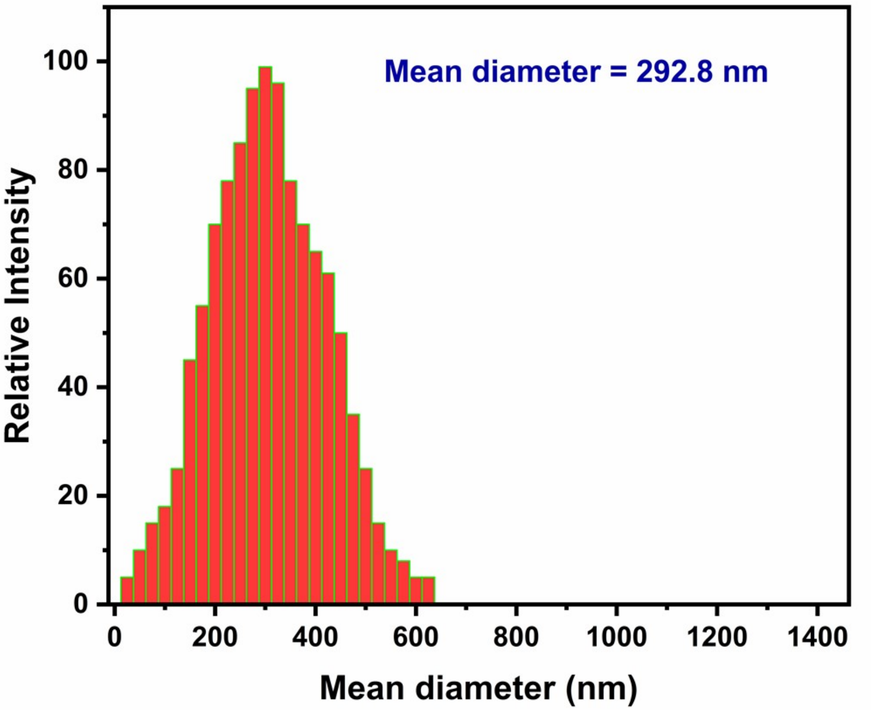

Fig. S20 DLS data profile in 90% H_2O/THF fraction: Size distribution patterns for **CA1.**

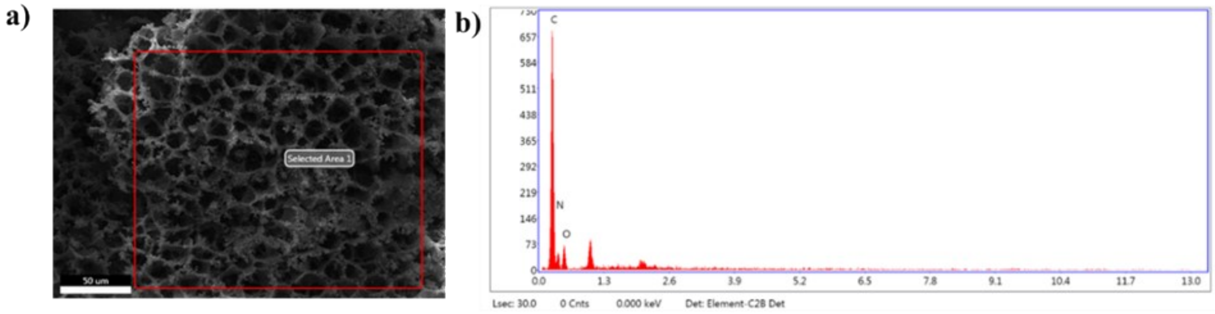

c)

eZAF Smart Quant Results

Element	Weight %	Atomic %	Net Int.	Error %	Kratio	Z	R	A	F
C K	54.69	59.92	125.98	5.78	0.3926	1.0151	0.9935	0.7073	1.0000
N K	24.08	22.63	8.58	19.50	0.0245	0.9907	1.0032	0.1027	1.0000
O K	21.23	17.46	13.03	17.12	0.0236	0.9696	1.0119	0.1149	1.0000

Fig. S21 a) selected area of EDAX for **CA1**; b) and c) elemental composition by EDAX for **CA1**.

a) b)

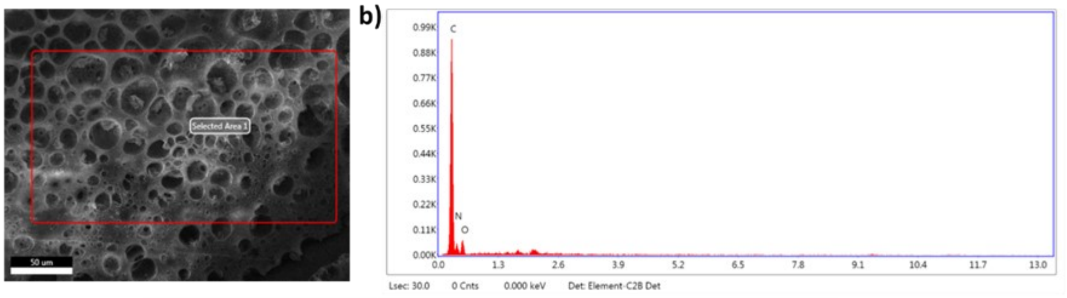

c)

eZAF Smart Quant Results

Element	Weight %	Atomic %	Net Int.	Error %	Kratio	Z	R	A	F
C K	59.57	64.52	192.07	5.13	0.4445	1.0132	0.9943	0.7362	1.0000
N K	22.54	20.94	9.95	18.85	0.0211	0.9889	1.0039	0.0946	1.0000
O K	17.88	14.54	14.32	16.78	0.0193	0.9678	1.0126	0.1114	1.0000

Fig. S22 a) selected area of EDAX for **CA1** ⊃ TPE; b) and c) elemental composition by EDAX for **CA1** ⊃ TPE.

a) b)

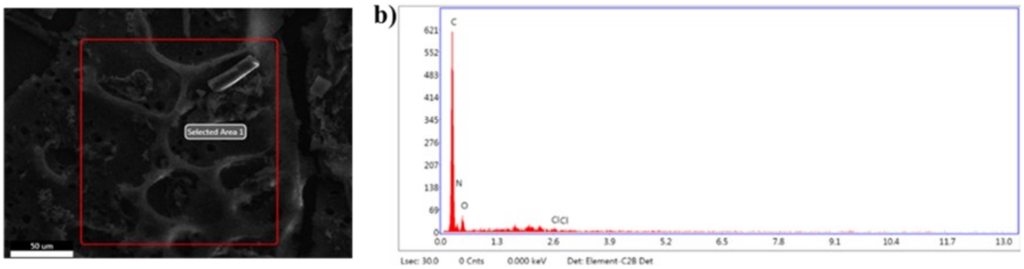

c)

eZAF Smart Quant Results

Element	Weight %	Atomic %	Net Int.	Error %	Kratio	Z	R	A	F
C K	64.22	69.31	117.08	6.00	0.4498	1.0130	0.9942	0.6917	1.0000
N K	16.71	15.47	4.09	27.09	0.0144	0.9887	1.0039	0.0871	1.0000
O K	18.54	15.02	9.25	18.45	0.0207	0.9676	1.0126	0.1153	1.0000
ClK	0.54	0.20	1.87	64.27	0.0046	0.8163	1.0666	1.0181	1.0243

Fig. S23 a) selected area of EDAX for **CA1** ⊃ TPE@RhB; b) and c) elemental composition by EDAX for **CA1** ⊃ $TPE@RhB$.

Table S1 Fluorescence emission maxima in different solvents.

**Solvents**	**CA1** ⊃ TPE ($\boldsymbol{\lambda_{emission}}$ in nm)
**Hexane**	405
**EA**	430
**MeOH**	427
**$CHCl_3$**	418
**THF**	420
**ACN**	447
**DMF**	413
**DMSO**	440

Table S2 Fluorescence lifetime parameters.

**Systems**	τ_1 **(ns) [B1]**	τ_2**(ns) [B2]**	χ^2	τ_{av} **(in ns)**

**CA+TPE**	2.16 [71.26]	3.63 [28.74]	1.01	2.58
**CA1+TPE+RhB**	1.69 [62.39]	2.90 [37.61]	1.08	2.14

Table S3 Energy transfer efficiency for **CA1 ⊃ TPE** and **CA1 ⊃ TPE@RhB**.

LHSs	λ_{ex}	I_{DA}	I_{D}	$\Phi_{ET}(\%)$
**CA1 ⊃ TPE+RhB**	320	2595.91	23569.98	88.98%

Table S4 Antenna effect for CA1 ⊃ TPE@RhB

LHSs	$I_{A+D(\lambda_{ex}=320\,nm)}^{577\,nm}$	$I_{D(\lambda_{ex}=320\,nm)}^{577\,nm}$	$I_{A+D(\lambda_{ex}=520\,nm)}^{577\,nm}$	Antenna effect
**CA1** ⊃ TPE+RhB	56619.92	9552.49	4238.906	11.1

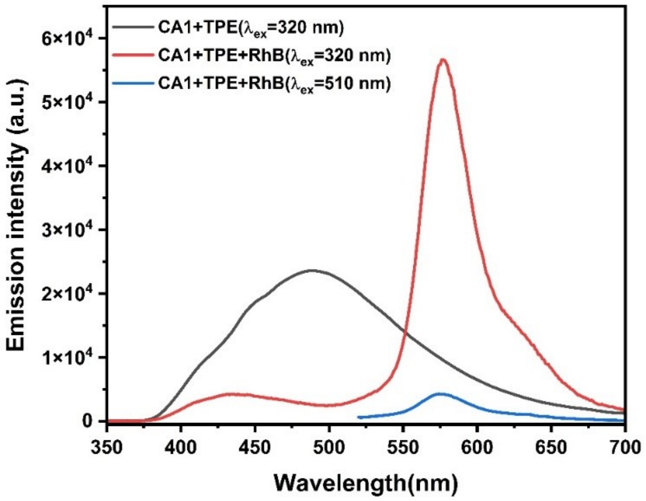

Fig. S24 Fluorescence emission intensity of **CA1** ⊃ TPE (λ_{ex} = 320 nm, 10^{-5} M [**CA1**⊃TPE]), **CA1** ⊃ TPE +RhB (λ_{ex} = 320 nm, c = 10^{-5} M [**CA1** ⊃ TPE] and c =10^{-6} M [RhB]), and RhB{ λ_{ex} = 510 nm, [RhB] = 10^{-6}M}.

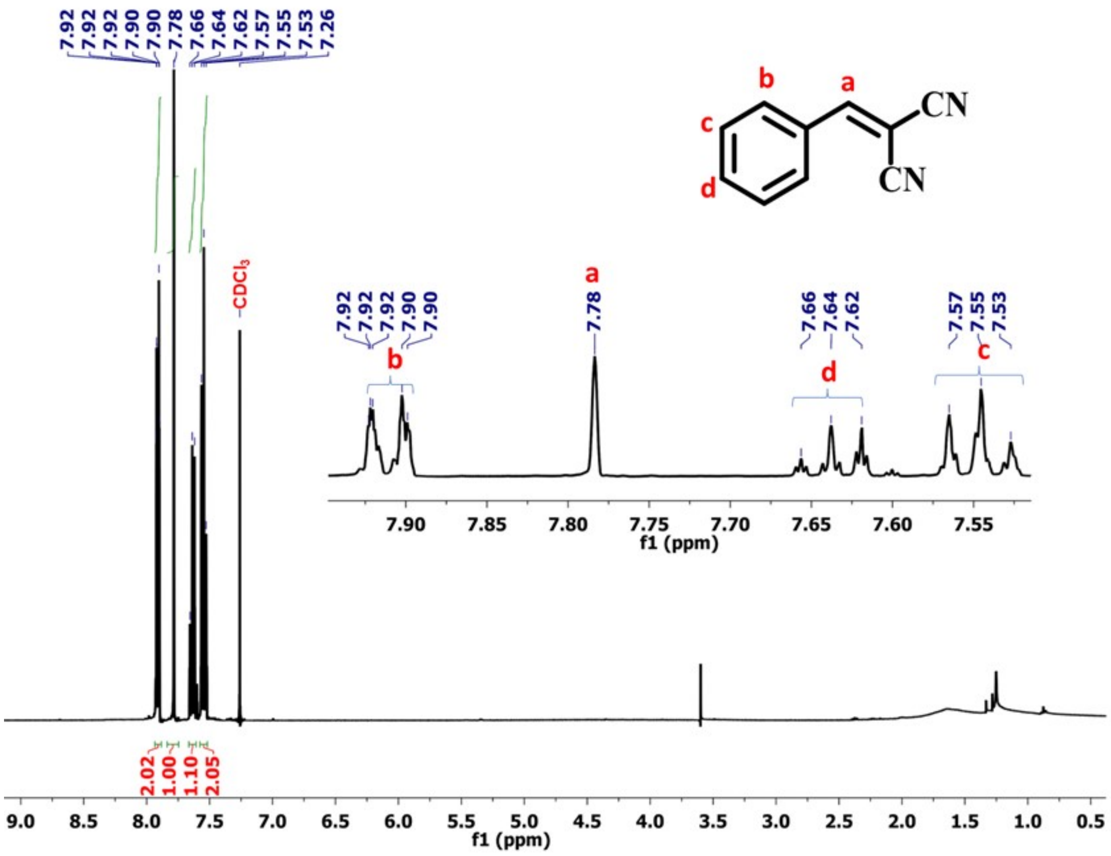

Fig. S25 ^{1}H NMR ($CDCl_3$, 400 MHz) of **7a**.

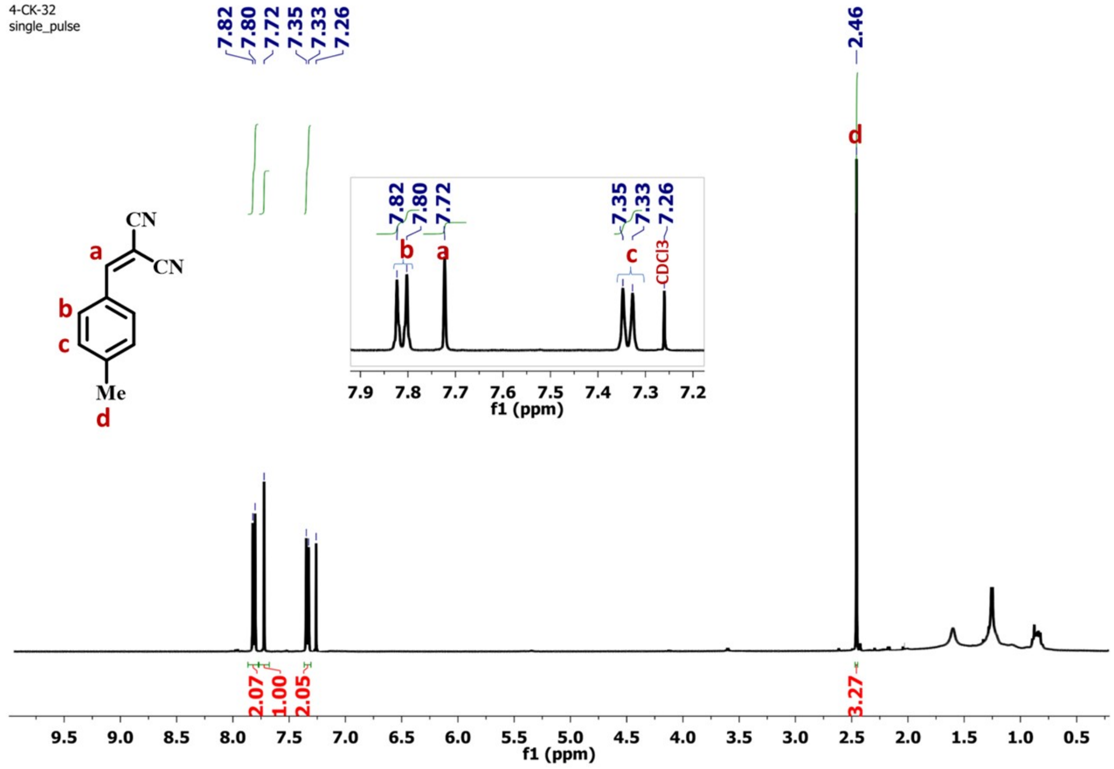

Fig. S26 ^{1}H NMR ($CDCl_3$, 400 MHz) of **7b**.

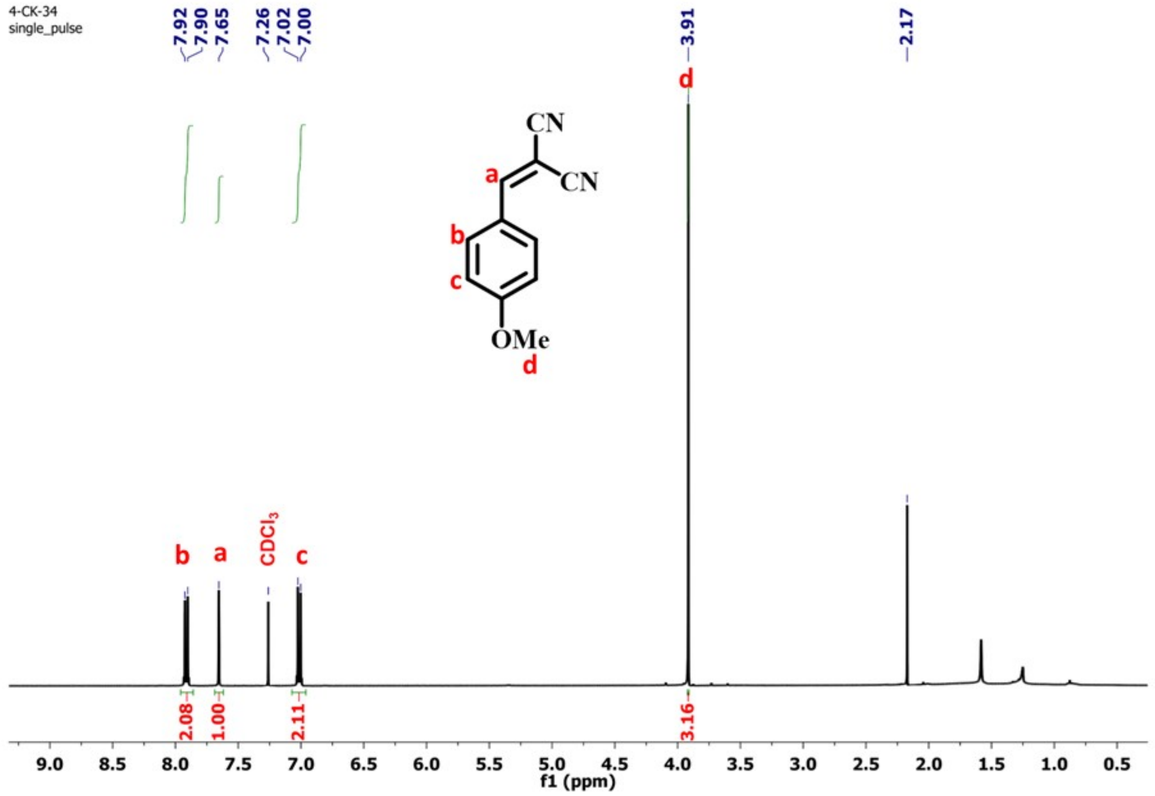

Fig. S27 ^{1}H NMR ($CDCl_3$, 400 MHz) of **7c**.

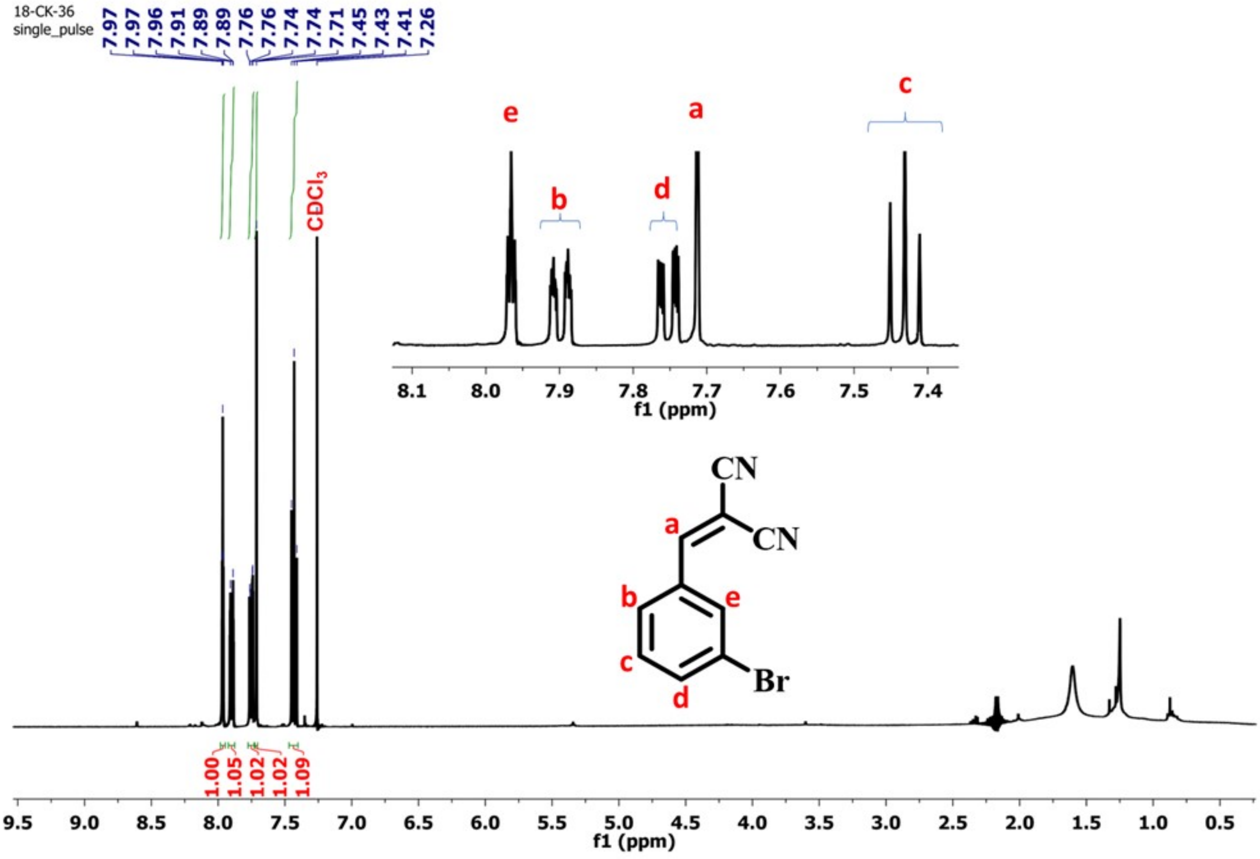

Fig. S28 ^{1}H NMR ($CDCl_3$, 400 MHz) of **7d**.

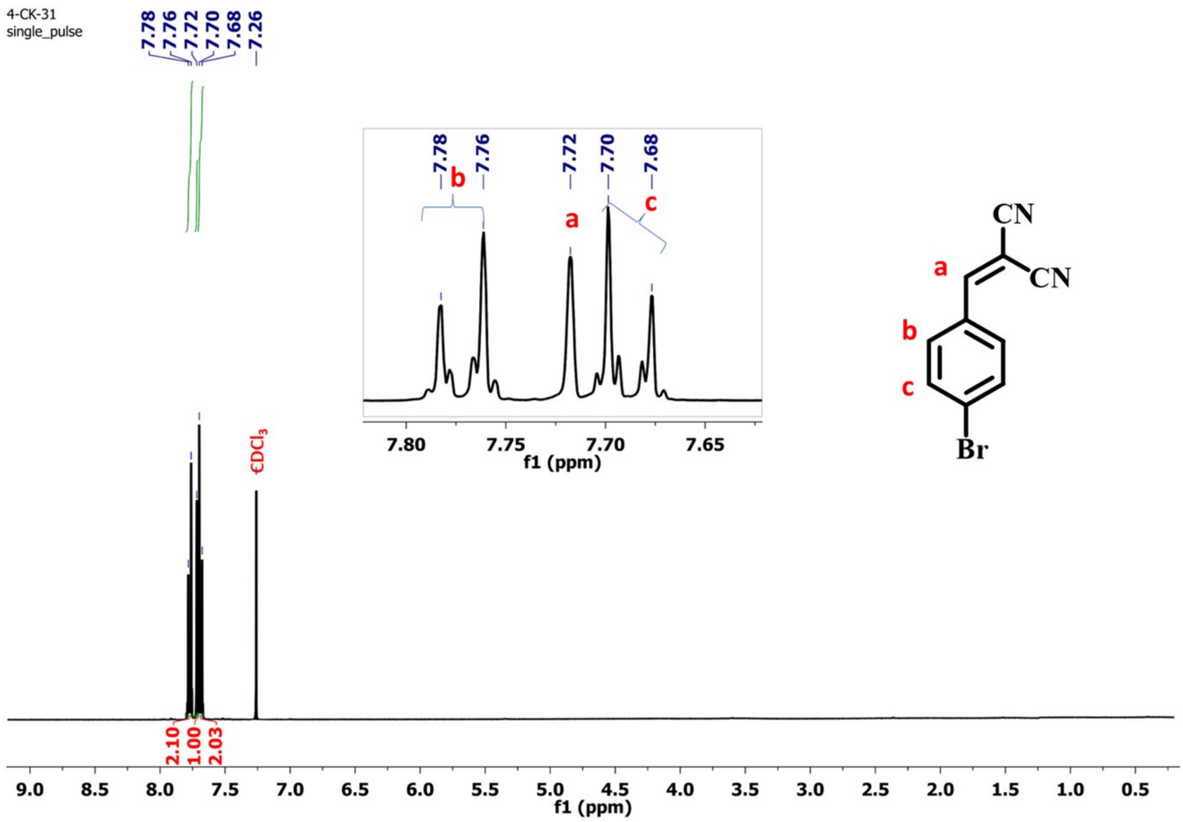

Fig. S29 ^{1}H NMR ($CDCl_3$, 400 MHz) of **7e**.

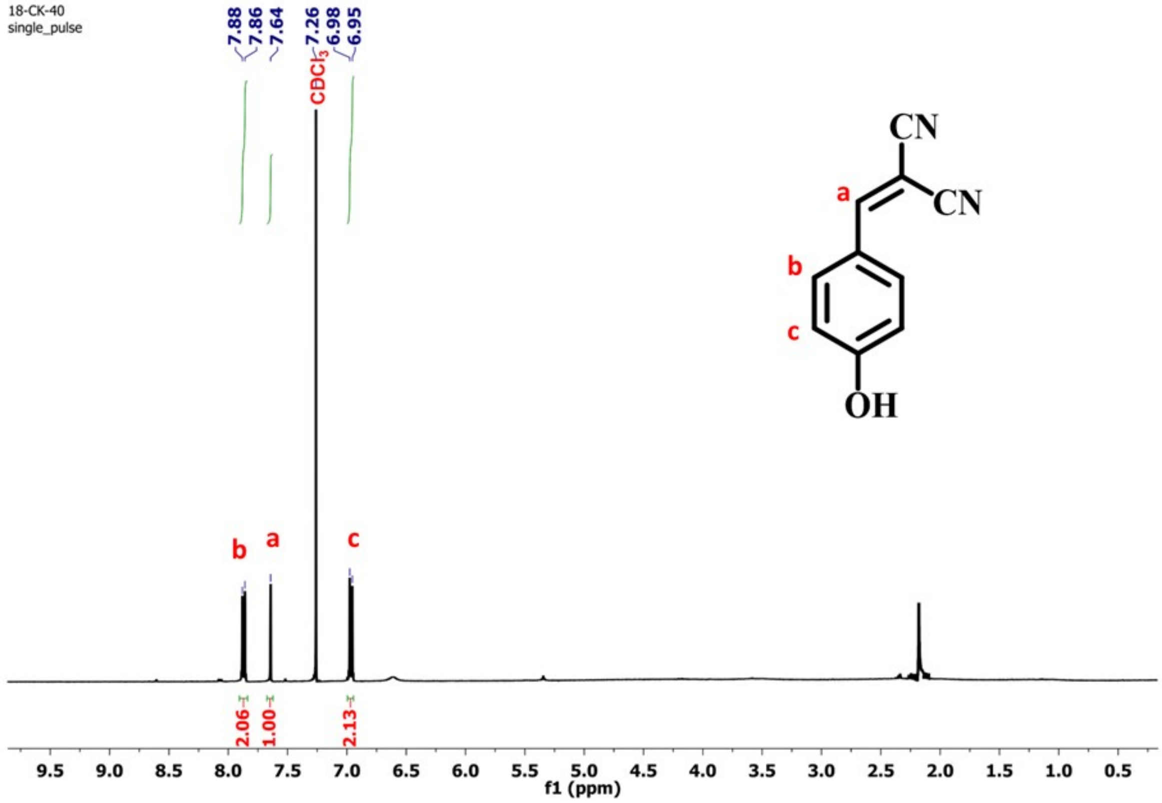

Fig. S30 ^{1}H NMR ($CDCl_3$, 400 MHz) of **7f**.

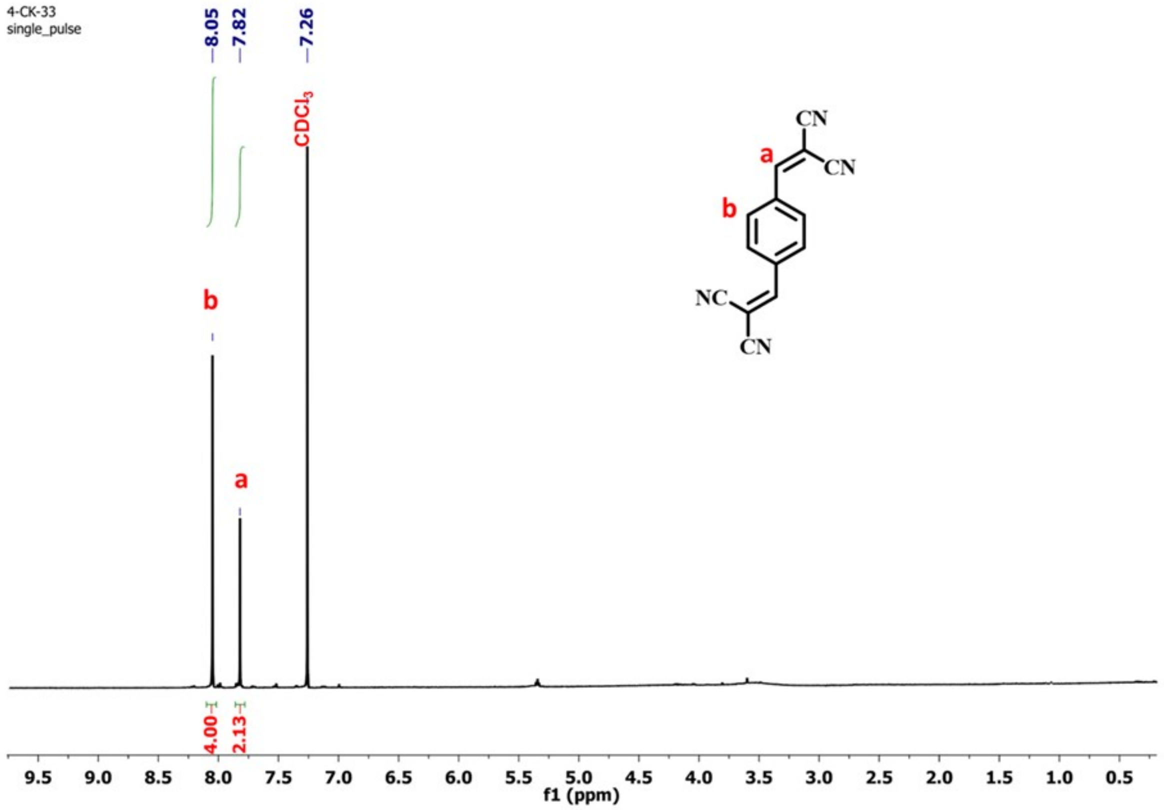

Fig. S31 ^{1}H NMR ($CDCl_3$, 400 MHz) of **7g**.

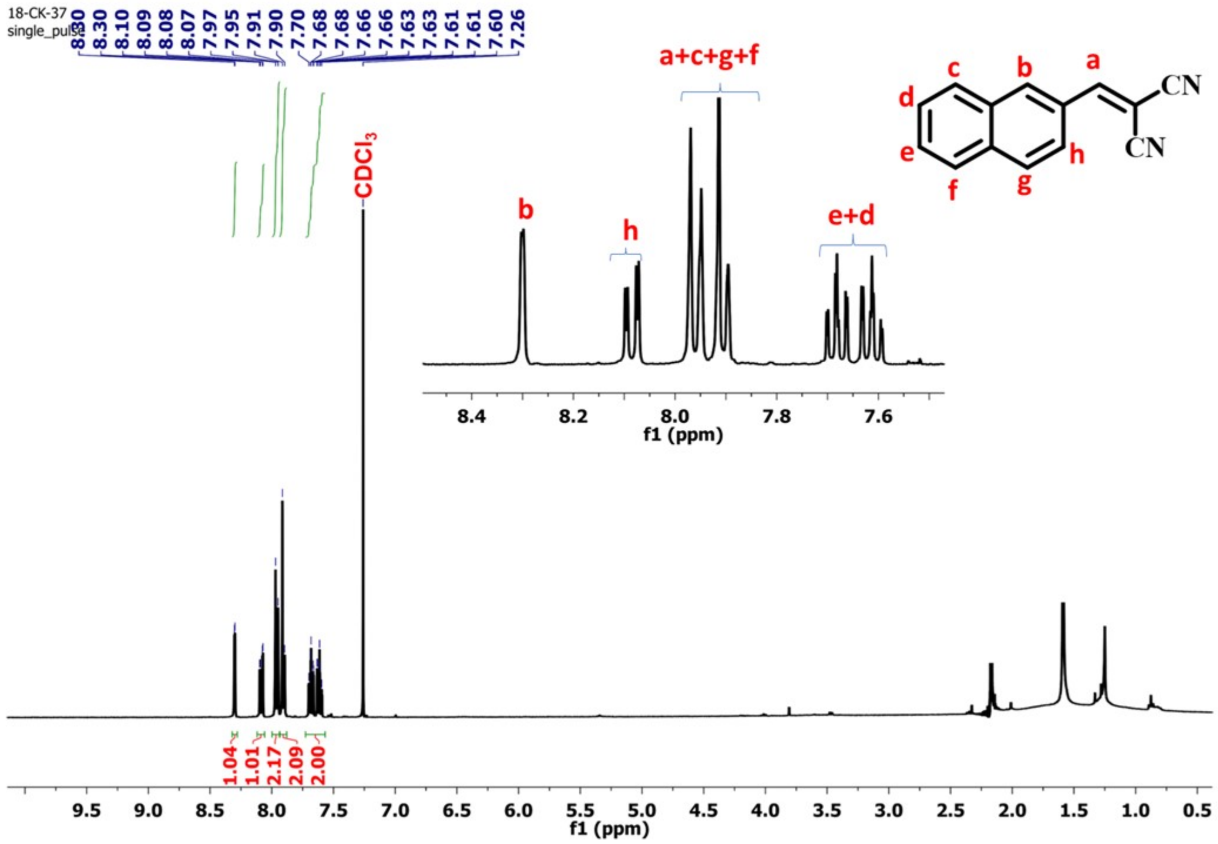

Fig. S32 ^{1}H NMR ($CDCl_3$, 400 MHz) of **7h**.

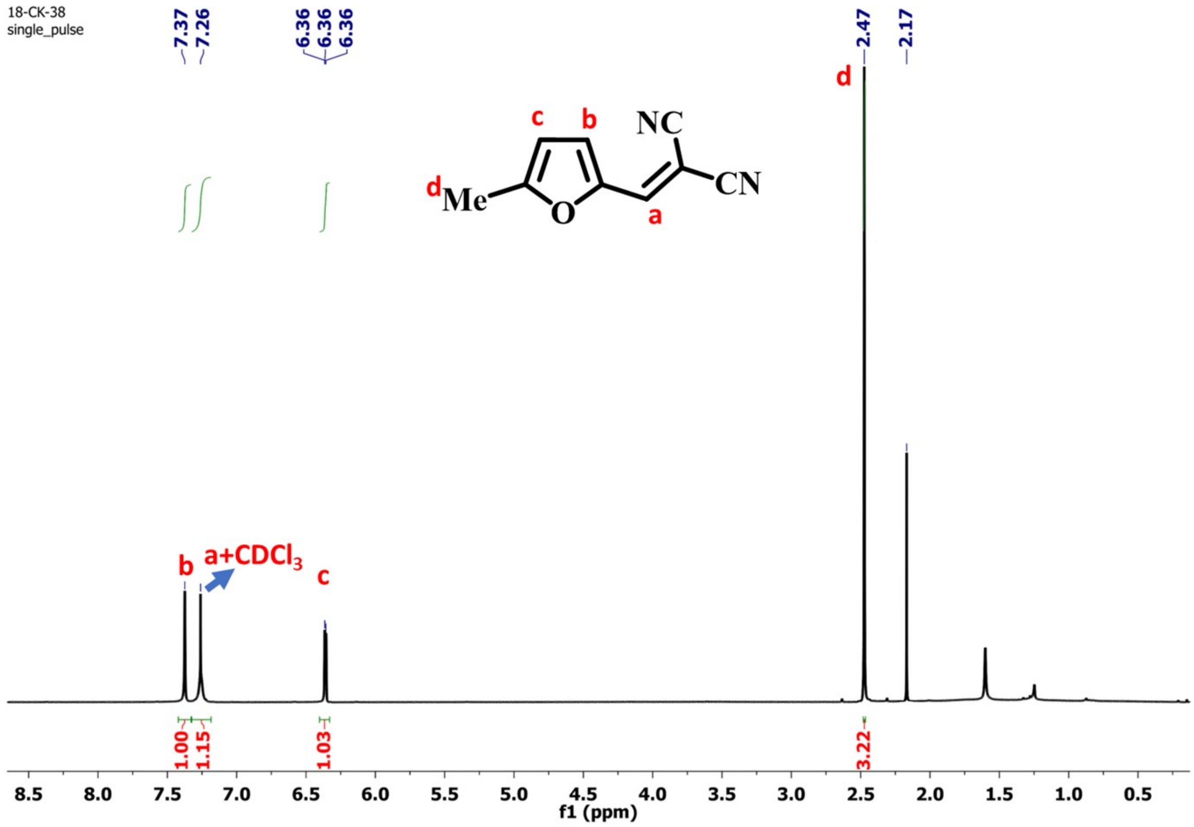

Fig. S33 ^{1}H NMR ($CDCl_3$, 400 MHz) of **7i**.

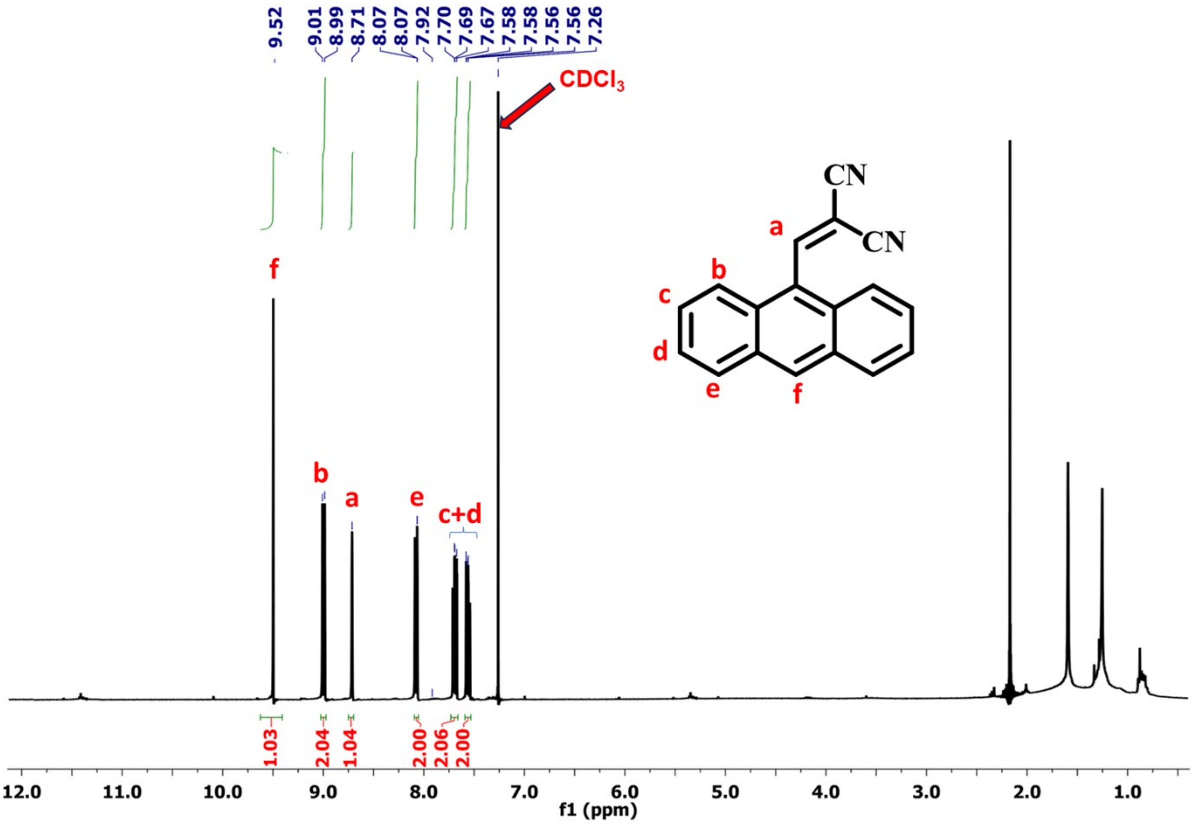

Fig. S34 ^{1}H NMR ($CDCl_3$, 400 MHz) of **7j**.

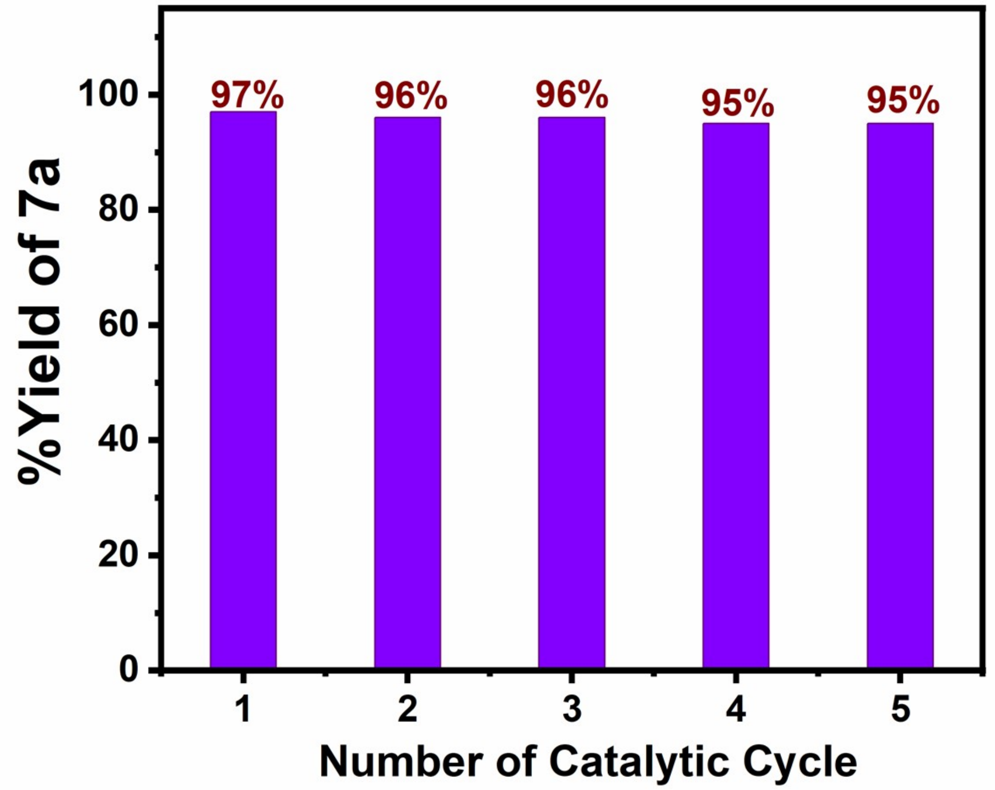

Fig. S35 Number of catalytic cycles by **CA1** ⊃ TPE@RhB for the formation of product **7a**.

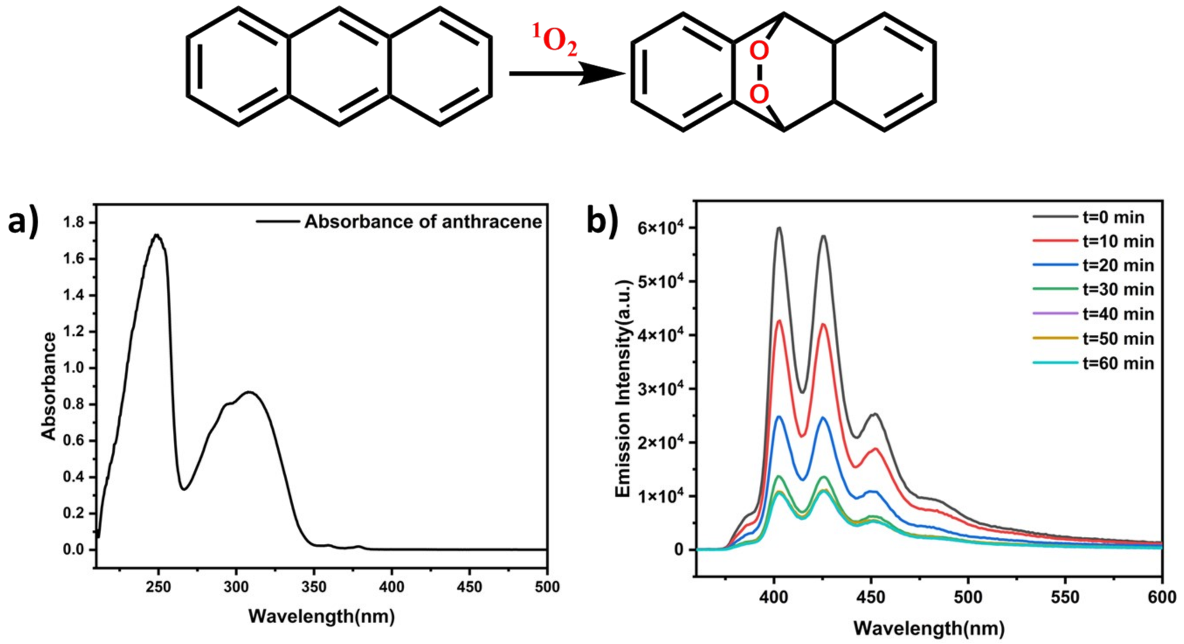

Fig. S36 a) UV-Visible spectra of 10^{-5} M anthracene in THF; b) Fluorescence emission titration of anthracene (conc. 10^{-5}M, λ_{ex} = 310 nm) with the 10 μL reaction mixture* at different time interval. (*Benzaldehyde **5a** (0.97 mmol), Malononitrile **6** (1.95 mmol), water (3ml), **CA1** ⊃ TPE (7.5 mol%), RhB (1.5 mol%).

Table S5. Detection of H_2O_2 generated during (photocatalysis reaction) starch/potassium iodide (KI) indicator

**3 mL solution under visible light**	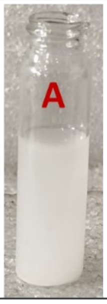	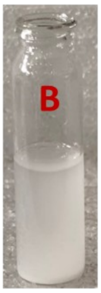	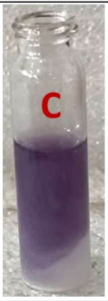	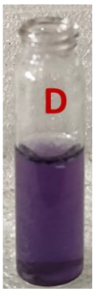	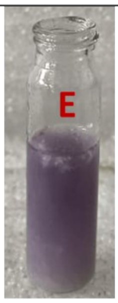	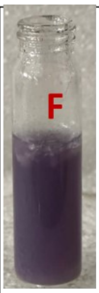	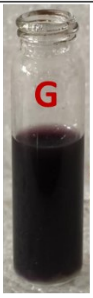
solution	Reaction mixture[a] at t=0	Starch+KI solution in water[b]	Addition of 0.2 mL solution B to the reaction mixture[a] at t=15 min	Addition of 0.2 mL solution B to the reaction mixture[a] at t=30 min	Addition of 0.2 mL solution B to the reaction mixture[a] at t=45 min	Addition of 0.2 mL solution B to the reaction mixture[a] at t=60 min.	Addition of 0.2 mL H_2O_2 to the solution B.

[a]Reaction mixture: Benzaldehyde **5a** (0.97 mmol), Malononitrile **6** (1.95 mmol), water (3ml), **CA1** ⊃ TPE (7.5 mol%), RhB (1.5 mol%) ; [b]Solution of Starch (0.2 mmol) +KI (0.2 mmol) Solution in 3 ml water.

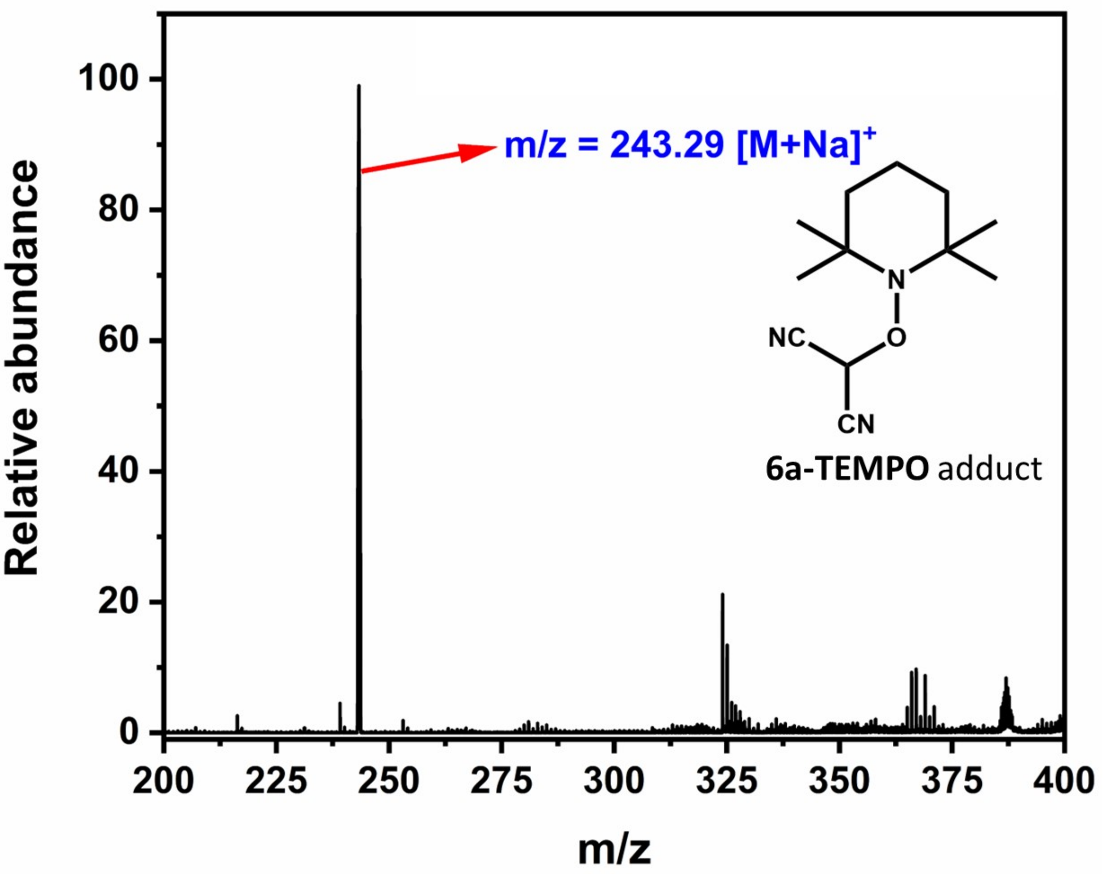

Fig. S37. ESI-MS spectra of **6a**-TEMPO adduct.

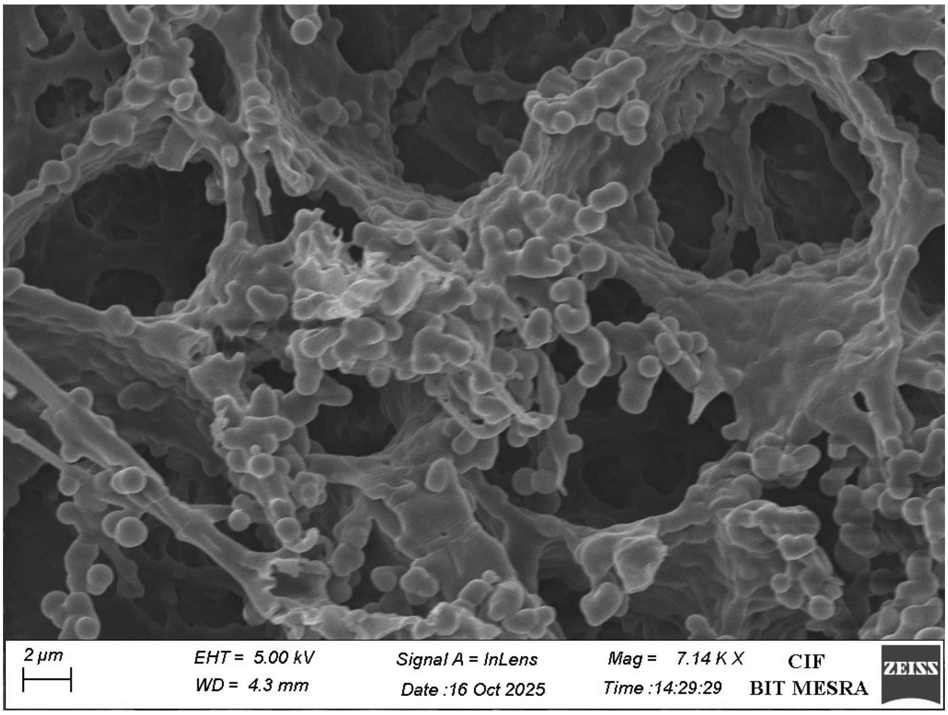

Fig. S38 a) FESEM image for recovered **CA1** ⊃ TPE@RhB after the catalysis reaction.

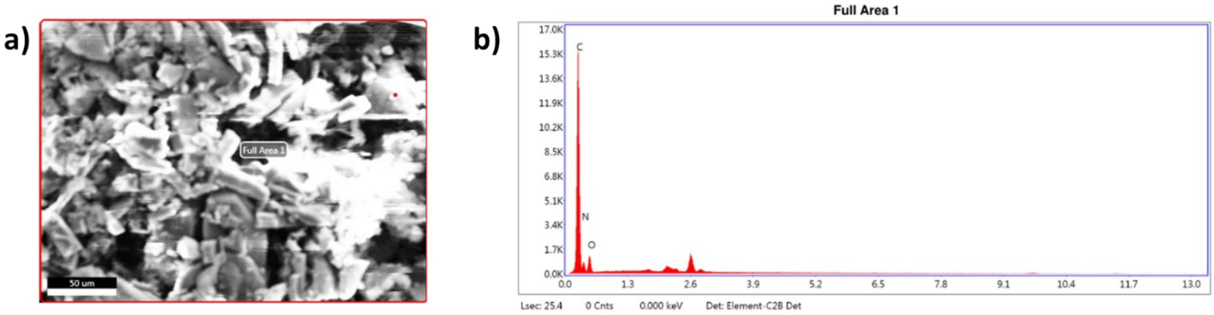

c)

eZAF Smart Quant Results

Element	Weight %	Atomic %	Net Int.	Error %	Kratio	Z	R	A	F
C K	59.37	64.43	3671.74	3.97	0.4383	1.0136	0.9941	0.7285	1.0000
N K	21.17	19.71	181.67	12.37	0.0199	0.9892	1.0038	0.0949	1.0000
O K	19.46	15.86	309.55	11.16	0.0215	0.9682	1.0125	0.1142	1.0000

Fig. S39 a) selected area of EDAX; b) and c) elemental composition by EDAX for recovered **CA1** ⊃ TPE@RhB after the catalysis reaction.
